# The Anticancer, Antioxidant and Antimicrobial Properties of the Sesquiterpene β-Caryophyllene from the Essential Oil of *Aquilaria crassna*

**DOI:** 10.3390/molecules200711808

**Published:** 2015-06-26

**Authors:** Saad S. Dahham, Yasser M. Tabana, Muhammad A. Iqbal, Mohamed B. K. Ahamed, Mohammed O. Ezzat, Aman S. A. Majid, Amin M. S. A. Majid

**Affiliations:** 1 EMAN Research and Testing Laboratory, School of Pharmaceutical Sciences, Universiti Sains Malaysia, 11800 Minden, Pulau Pinang, Malaysia; E-Mails: hawk_dijla@yahoo.com (S.S.D.); yasser.tabana@hotmail.com (Y.M.T.); 2 School of Chemical Sciences, Universiti Sains Malaysia, 11800 Minden, Pulau Pinang, Malaysia; E-Mail: adnan_chem38@yahoo.com; 3 EMAN Biodiscoveries Sdn. Bhd. EUREKA Complex, Universiti Sains Malaysia (USM) Campus, 11800 Minden, Pulau Pinang, Malaysia; E-Mail: khadeer.nc@gmail.com; 4 Centre for Drug Research, Universiti Sains Malaysia, 11800 Minden, Pulau Pinang, Malaysia; E-Mail: mohamed_oday@yahoo.com; 5 Advanced Medical and Dental Institute (IPPT), Universiti Sains Malaysia, Bertam, 13200 Kepala Batas, Penang, Malaysia

**Keywords:** β-caryophyllene, anti-cancer, apoptosis, anti-clonogenic, nuclear fragmentation, colorectal cancer

## Abstract

The present study reports a bioassay-guided isolation of β-caryophyllene from the essential oil of *Aquilaria crassna*. The structure of β-caryophyllene was confirmed using FT-IR, NMR and MS. The antimicrobial effect of β-caryophyllene was examined using human pathogenic bacterial and fungal strains. Its anti-oxidant properties were evaluated by DPPH and FRAP scavenging assays. The cytotoxicity of β-caryophyllene was tested against seven human cancer cell lines. The corresponding selectivity index was determined by testing its cytotoxicity on normal cells. The effects of β-caryophyllene were studied on a series of *in vitro* antitumor-promoting assays using colon cancer cells. Results showed that β-caryophyllene demonstrated selective antibacterial activity against *S. aureus* (MIC 3 ± 1.0 µM) and more pronounced anti-fungal activity than kanamycin. β-Caryophyllene also displayed strong antioxidant effects. Additionally, β-caryophyllene exhibited selective anti-proliferative effects against colorectal cancer cells (IC_50_ 19 µM). The results also showed that β-caryophyllene induces apoptosis via nuclear condensation and fragmentation pathways including disruption of mitochondrial membrane potential. Further, β-caryophyllene demonstrated potent inhibition against clonogenicity, migration, invasion and spheroid formation in colon cancer cells. These results prompt us to state that β-caryophyllene is the active principle responsible for the selective anticancer and antimicrobial activities of *A. crassnia*. β-Caryophyllene has great potential to be further developed as a promising chemotherapeutic agent against colorectal malignancies.

## 1. Introduction

*Aquilaria crassna* (Thymelaeaceae) has been used in diverse Chinese and Southeast Asian traditional medicine systems to treat infectious and inflammatory diseases, arthritis and cardiac disorders. In the Middle East this woody plant has been widely used in fragrances, incense and cosmetics. The plant, locally known in Indonesia as gaharu or agarwood, contains biologically active essential oils which have been used for various medicinal purposes by a number of civilizations due to their phytochemically rich and pharmacologically active aromatic compounds [1]. It has been widely used by Arabs and Japanese to treat digestive, neurodegenerative and sedative disorders [2,3]. In Thailand, *A. crassna* extract known as one of the ingredients of “Ya-hom”, a traditional Thai herbal formulation for the treatments of various disorders including inflammation, aging, cancer and cardiovascular disorders [4,5].

A number of scientific studies have revealed that the essential oil extracts of *A. crassna* possess antioxidant, antimicrobial, cytotoxic, antipyretic, analgesic, anti-ischemic, laxative and digestive effects [6,7,8,9,10,11]. The most important bioactive constituents of the plant are alkaloids, tannins, flavonoids and phenolic compounds [12,13]. Several scientific studies have showed the presence of a versatile class of polyphenols in *A. crassna*. The major polyphenols in the plant are glycosides of flavonoids, benzophenones, and xanthones. Among the active principles identified were iriflophenone 3,5-*C*-β-diglucoside, iriflophenone 3-*C*-β-glucosidemangiferin, iriflophenone 2-*O*-α-rhamnoside, genkwanin 5-*O*-β-glucoside, and genkwanin 4′-methyl ether 5-*O*-β-primeveroside [14,15,16]. Sesquiterpenes are reported to be the main active compounds in agarwood and were thought to be the active principles of the plant [17].

In the present study, an essential oil mixture from *A. crassna* bark was subjected to bioactivity-guided fractionation and repeated column chromatography to afford β-caryophyllene as an active principle. The identity of the β-caryophyllene was elucidated by physicochemical spectral studies. β-Caryophyllene was then tested for its inhibitory effect on proliferation of a panel of human cancer and normal cell lines. In addition, the antimicrobial effect of β-caryophyllene was tested against some human pathogens. Finally, to elucidate the mechanism of action and to characterize the mode of cytotoxicity induced by β-caryophyllene in human colorectal cancer cells, a series of *in vitro* assays, such as Hoechst 33342, rhodamine 123, colony formation, migration and invasion assays were performed.

## 2. Results and Discussion

### 2.1. Extraction of Essential Oils and Isolation of the Active Principle

**Figure 1:**
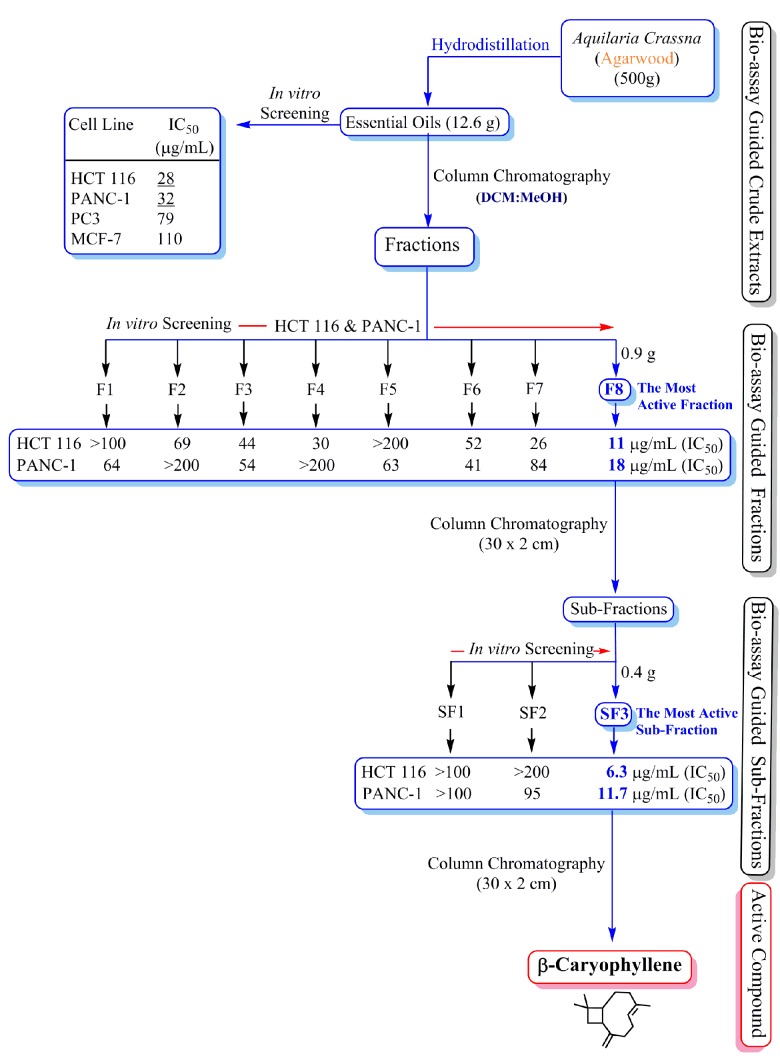
Schematic diagram showing the bioassay (anti-proliferative assay)-guided isolation of β-caryophyllene from the essential oils of *Aquilaria crassna*.

*A. crassna* is a plant with diverse traditional medicinal properties. A number of studies have confirmed that the essential oil is an active component of *A. crassna* stem bark [18,19], but very little is known about the active principle(s) responsible for the pharmacological properties of the plant. The present study clearly demonstrated that the essential oil extracted from *A. crassna* using the hydrodistillation method showed remarkable antiproliferative properties against human colorectal cancer cells. The main advantage of the hydrodistillation method over steam distillation is that less steam is used hence a shorter processing time is required and therefore the method gives a high yield of oil. This method is also less harsh on the botanical material and therefore the biological efficacy of the phytochemicals will be retained. Using repeated column chromatography on the essential oil, its fractions and subfractions were obtained, whereas the bioassay-guided screening resulted in the identification of the active principle of the essential oils (Figure 1). The chemical structure of the active principle was elucidated using spectroscopic data that confirmed it was β-caryophyllene in nearly pure form (see Supporting Information).

In the present study, the powdered material (500 g) of *A. crassna* stem bark was subjected to hydrodistillation to obtain the essential oil in a yield of 2.52%. The essential oil mixture showed significant anti-proliferation activity against HCT 116 (IC_50_ 28 µg·mL^−1^), PANC-1 (IC_50_ 32 µg·mL^−1^), PC3 (IC_50_ 79 µg·mL^−1^) and MCF-7 (IC_50_ 110 µg·mL^−1^) human cancer cell lines. The mixture of essential oils was subjected to column chromatography to obtain 12 fractions (F1-F12). Among all the fractions, fraction 8 (F8) showed most potent activity (HCT 116 IC_50_ 11 µg·mL^−1^; PANC-1 IC_50_ 18 µg·mL^−1^; PC3 IC_50_ 26 µg·mL^−1^ and MCF-7 IC_50_ 72 µg·mL^−1^). Further chromatographic separation of the fraction 8 yielded three sub-fractions (SF1-SF3). Among the three sub-fractions, SF3 was found to be the most active one against the proliferation of HCT 116 (IC_50_ 6.3 µg·mL^−1^) and PANC-1 (IC_50_ 11.7 µg·mL^−1^). Recrystallization of sub-fraction SF3 using hot methanol yielded β-caryophyllene.

### 2.2. Characterization of A. crassna Essential Oil and β-Caryophyllene Using GC-MS

**Figure 2:**
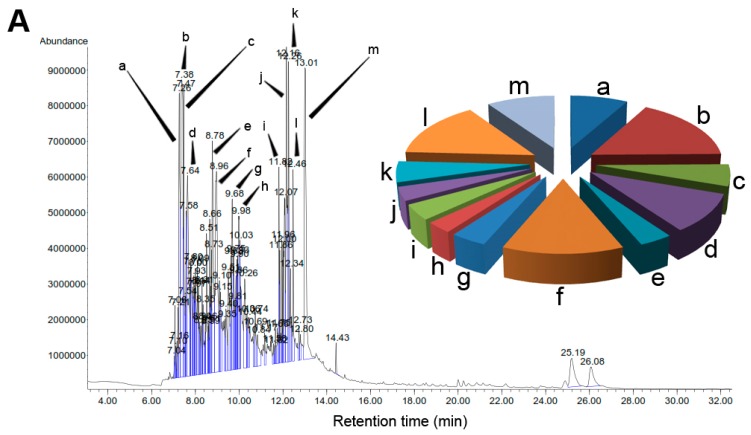

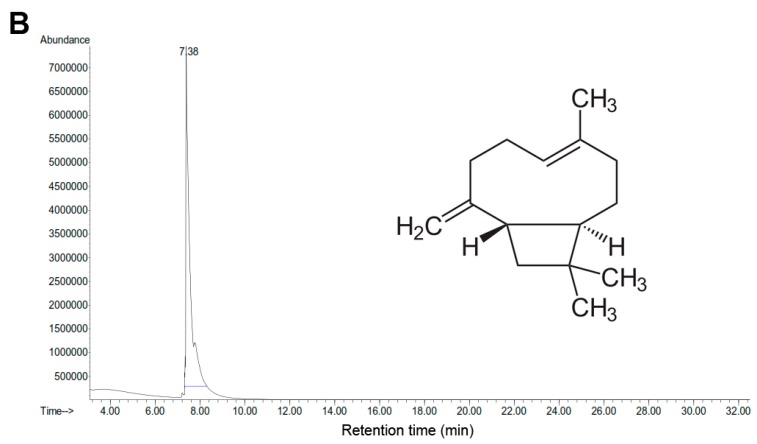
Gas chromatograpic analysis of *Aquilaria crassna* and β-caryophyllene. (**A**) Chemical characterization of the essential oil of *Aquilaria crassna* by GC-MS. The pie charts depict the relative chemical composition of the sub-fractions. Refer to Table S1 in the Supporting Information for the details of the peaks identified in the chromatogram; (**B**) The major peak corresponds to the the active principle, identified as β-caryophyllene, isolated from the essential oil of *Aquilaria crassna.*

*A. crassna* essential oil and the isolated compound β-caryophyllene were subjected to GC-MS analysis to quantify the major chemical constituents and confirm their molecular weights, respectively. Figure 2 depicts the comparative GC-MS (Figure 2A) of the extract and β-caryophyllene (Figure 2B). The pie chart in Figure 2A represents the composition and proportion of the major phytoconstituents present in *A. crassna* essential oil. The GC-MS data such as retention time (R_t_), % area peak, molecular formula and molecular weight obtained for the major chemical components are given in the Supporting Information (Table S1). In addition, the mass spectra for all the major chromatographic peaks identified are given in the Supporting Information (Figure S1A–M) are given in Table S1. The chemical composition of the essential oil was identified using the NIST library. Accordingly, the GC-MS analysis revealed that the essential oil mixture was composed of various polyphenols and aromatic compounds. The quantitative analysis of the essential oil showed that the major peaks corresponded to β-caryophyllene (8.1%), 1-phenanthrenecarboxylic acid (7.1%), 2-naphthalene-methanol (6.2%), α-caryophyllene (4.7%), benzenedicarboxylic acid (4.6%), azulene (3.9%), naphthalene (2.7%) and cyclodecene (2.6%).

### 2.3. Antioxidant Activity

The radical scavenging capability of β-caryophyllene, determined by the DPPH and FRAP scavenging methods is depicted in Table 1. The results of the current study are in agreement with a previous study on the antioxidant efficacy of β-caryophyllene [20].

**Table 1:** Antioxidant efficacy of β-caryophyllene.

Sample	IC_50_ Values in μM	
	DPPH	FRAP
β-Caryophyllene	1.25 ± 0.06	3.23 ± 0.07
Ascorbic acid	1. 5 ± 0.03	3.8 ± 0.4

### 2.4. Antimicrobial Effect of β-Caryophyllene

β-Caryophyllene exhibited strong antibacterial effect against all the tested bacterial strains, with MIC values that ranged from 3 to 14 μM (Table 2). The compound showed more pronounced antibacterial activity against Gram-positive bacteria than Gram-negative bacteria (Table 2). The results revealed that *E. coli* was less susceptible than the other Gram-positive bacterial strains tested. The lowest MIC value was recorded against S. aureus (3 ± 0.4 µM).

**Figure 3:**
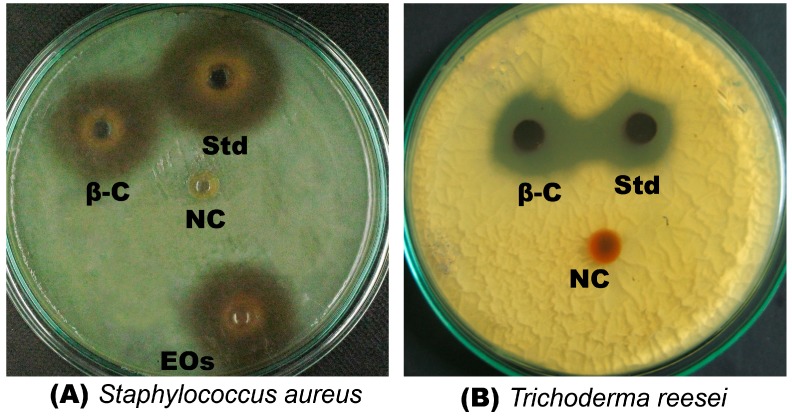
Antimicrobial effect of β-caryophyllene. (**A**) Antibacterial effect of β-caryophyllene (β-C) on *Staphylococcus aureus* (Std = Kanamycin; EOs = essential oil of *Aquilaria crassna*; NC = negative control); (**B**) Antifungal effect of β-caryophyllene (β-C) on *Trichoderma reesei* (Std = Kanamycin; NC = negative control).

The antifungal assay results indicates a significant activity of β-caryophyllene against all the tested fungi (Table 2). The activity of β-caryophyllene was more pronounced than the standard reference, kanamycin (Table 2). The antimicrobial activity of β-caryophyllene could be attributed to its strong antioxidant activities [21]. To the best of our knowledge, this is the first report on the antifungal activity of β-caryophyllene whereas, a previous study [22] reported the significant antibacterial effect of β-caryophyllene against *S. aureus*. In the present study, it is also demonstrated that β-caryophyllene significantly inhibits the growth of *S. aureus*, whereas it is ineffective against *K. pneumoniae*. Figure 3 depicts the antibacterial (Figure 3a) and antifungal (Figure 3b) activities of β-caryophyllene against *S. aureus* and *T. reesei*, respectively.

**Table 2:** Antimicrobial activity of β-caryophyllene.

Bacterial Strains	MTCC	Minimum Inhibitory Concentration (MIC) in μM	
		β-Caryophyllene	Kanamycin
*B. cereus*	1307	9 ± 1.1	2 ± 0.7
*B. subtilis*	6910	8 ± 2.1	4 ± 1.8
*S. aureus*	7405	3 ± 0.4	8 ± 2.3
*E. coli*	732	9 ± 2.2	7 ± 1.4
*K. pneumoniae*	7028	14 ± 2.7	2 ± 0.4
*P. aeruginosa*	4302	7 ± 1.2	9 ± 1.4
*A. niger*	2196	6 ± 0.8	7 ± 0.4
*P. citrinum*	7124	7 ± 1.2	9 ± 1.1
*R. oryzae*	1987	6 ± 0.5	7 ± 0.3
*T. reesei*	3929	4 ± 0.7	6 ± 1.4

### 2.5. Inhibitory Effect of β-Caryophyllene on the Proliferation of Cancer Cell Lines

The antiproliferative effect of β-caryophyllene was tested against seven tumor and two normal cell lines using the MTT assay. The median inhibitory concentration (IC_50_) values were calculated for each cell line and the values are presented in Table 3.

**Table 3:** IC_50_ values of β-caryophyllene on various human cancer cell lines.

Extracts	Carcinoma Cell Lines							Normal Cell Lines		
	HCT 116	PANC-1	HT-29	ME-180	PC3	K562	MCF-7	CCD-18Co	NIH/3T3-L1	RGC5
*A. crassna* (µg/mL)	28	32	82	98	79	142	110	579	133	98
β-Caryophyllene (µM)	19	27	63	95	104	105	285	612	530	156
Positive controls (µM)	5-FU ^a^	BA ^b^	5-FU	BA	BA	BA	Tam ^c^	BA	BA	BA
	12.7	19.4	15	37.1	8.4	17.3	9.5	62	53	97

^a^ 5-fluorouracil; ^b^ betulinic acid; ^c^ tamoxifen.

**Figure 4:**
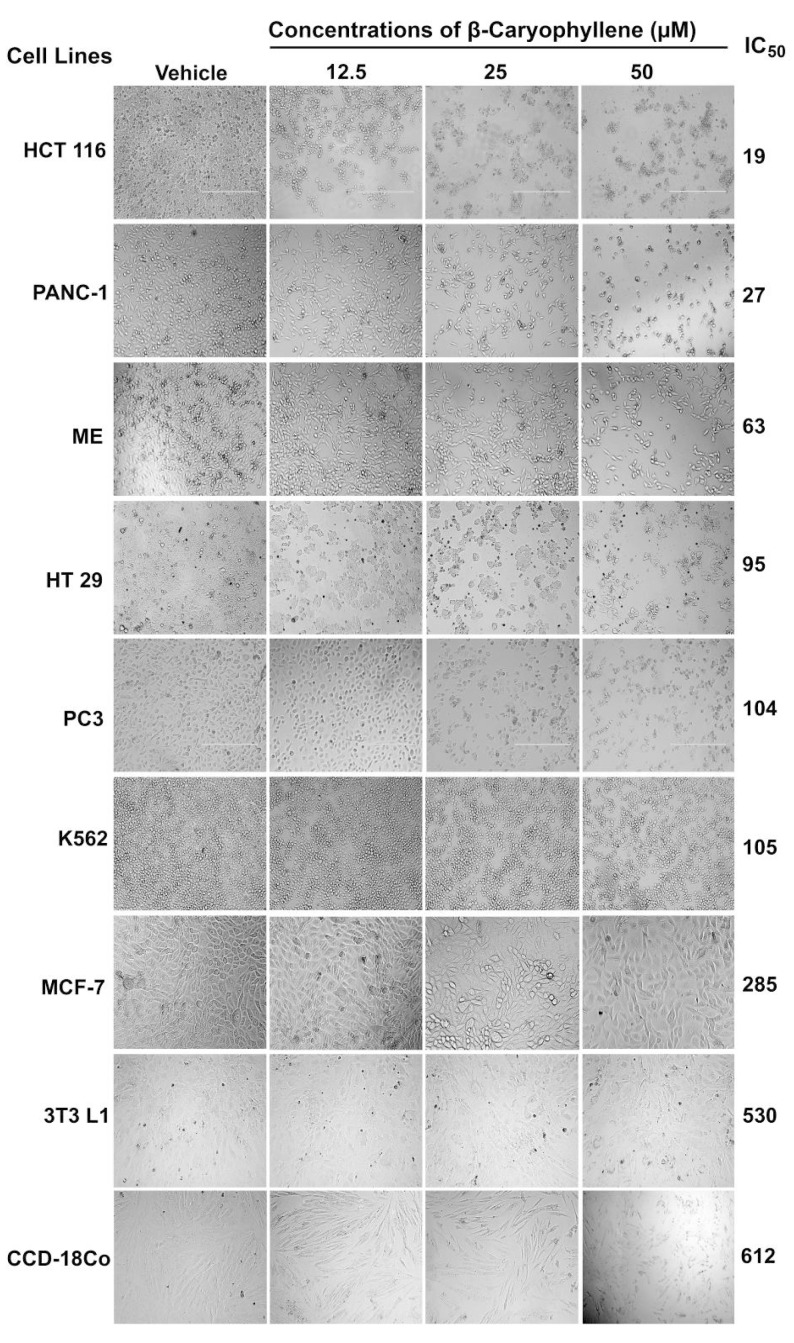
Effect of β-caryophyllene on the cellular morphology of human cancer and normal cell lines. Photomicrographic images of cancer cell lines, taken under an inverted phase-contrast microscope at 200× magnification using a digital camera at 48 h after treatment with β-caryophyllene.

**Figure 5:**
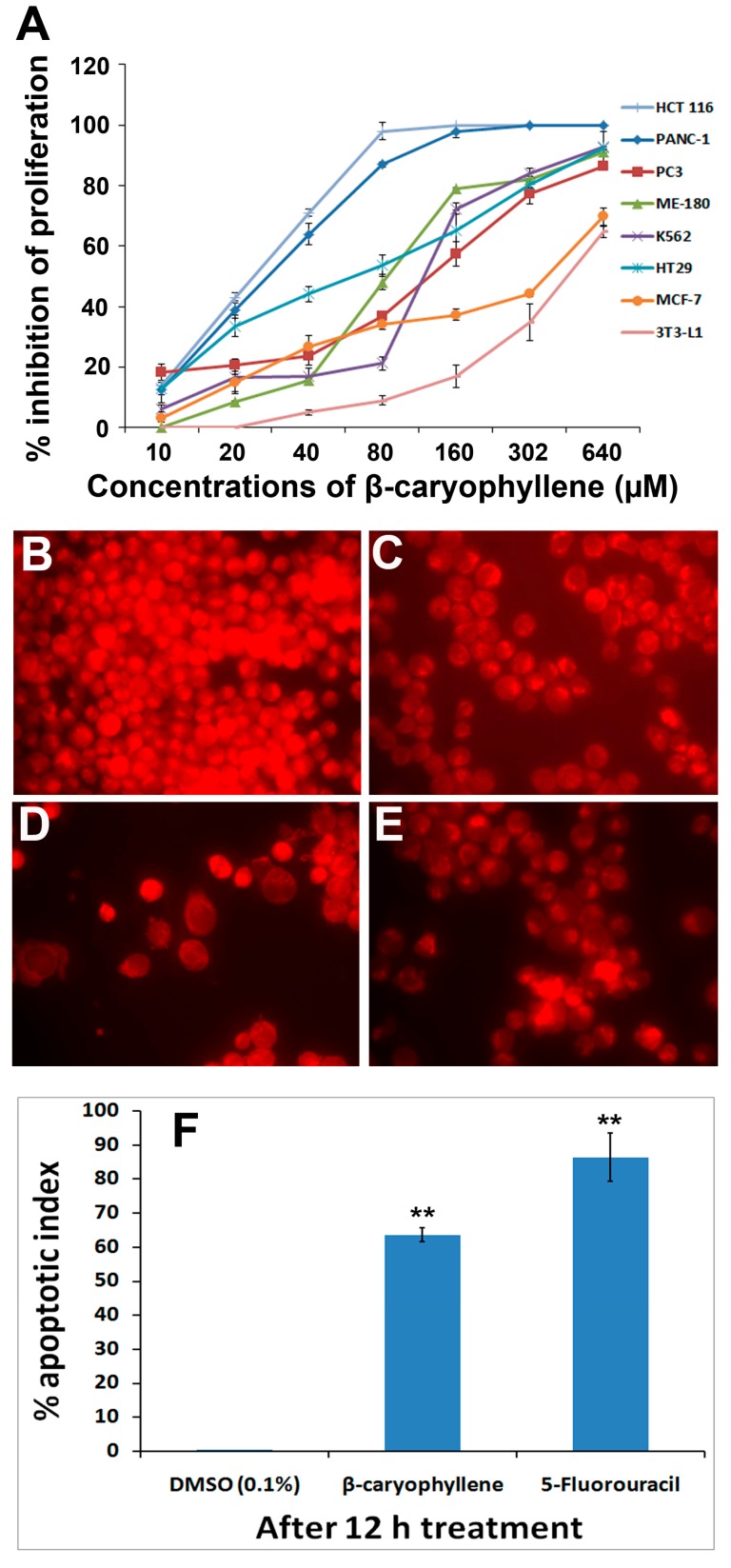
(**A**) Dose-dependent anti-proliferative effect of β-caryophyllene on, HCT 116, PANC-1, HT-29, MCF-7, PC3, K562, ME-180 and NIH/3T3-L1 cell lines was assessed by MTT-assay (values are represented as mean ± SD, *n* = 3); (**B**) Rhodamine 123 stained photomicrographic images of HCT 116 cells treated with vehicle (0.1% DMSO); (**C**) Rhodamine 123 stained photomicrographic images of HCT 116 cells treated with β-caryophyllene (10 µM) for 6 h; (**D**) Rhodamine 123 stained photomicrographic images of HCT 116 cells treated with β-caryophyllene (10 µM) for 12 h; (**E**) Rhodamine 123 stained photomicrographic images of HCT 116 cells treated with 5-flourouracil (10 µM) for 12 h; (**F**) Graphical representation of percentage of apoptotic indices. The apoptotic index for each test group was expressed as a percentage of the ratio of number of unstained cells to the total number of cell in 10 different microscopic fields. Values are presented as mean ± SD (*n* = 10), ** represents *p* < 0.01.

Among the tested cancer cells, the compound demonstrated selective anti-proliferative effect against three cancer cell lines, namely HCT 116 (colon cancer, IC_50_ = 19 µM), PANC-1 (pancreatic cancer, IC_50_ = 27 µM), and HT29 (colon cancer, IC_50_ = 63 µM) cells, whereas it exhibited either moderate or poor cytotoxic effects against ME-180, PC3, K562 and MCF-7. Noteworthily, the compound displayed low toxicity against the normal cell lines 3T3-L1 and RGC-5. The results were compared with the respective standard reference drugs, tamoxifen, betulinic acid and 5-fluorouracil. Figure 4 shows the effect of β-caryophyllene on the morphological structures of the cancer cell lines. The photomicrographic images revealed that β-caryophyllene did not affect the morphology of a normal cell line (3T3-L1), as the treated cells were more or less similar to the negative control, even for the higher concentration of β-caryophyllene. The selectivity index (SI) measures the selective cytotoxicity of a test sample against cancerous cells and the safety of sample towards normal cells. Compounds with a SI value of more than 3 are considered to have high selectivity towards the particular cancer cell line [23]. Table 4 presents the SI values of β-caryophyllene for various cancer cell lines tested. The analysis showed that β-caryophyllene possesses higher selectivity towards the colorectal cancer cells (HCT 116), with SI = 27.9, followed by PANC-1 and HT 29 cells with SI = 19.6 and 8, respectively. Therefore, in the present study further investigations on the cytotoxic effect of β-caryophyllene were carried out using HCT 116 cells. Figure 5A shows a graphical illustration of the dose-dependent antiproliferative effect of the compound on the panel of human cancer cell lines.

**Table 4:** Selectivity index of β-Caryophyllene which represents IC_50_ for normal cell line/IC_50_ for cancerous cell line.

Extracts	Selectivity Index (SI) ^a^						
	HCT 116	PANC-1	HT-29	ME-180	PC3	K562	MCF-7
β-Caryophyllene (µM)	32.2	22.7	9.7	6.4	5.9	5.8	2.1

^a^ Selective index = IC_50_ CCD-18Co cells/IC_50_ cancerous cells.

### 2.6. β-Caryophyllene Reduces Mitochondrial Membrane Potential in HCT-116 Cells

In order to obtain a deeper insight into an apoptotic effect of β-caryophyllene on mitochondrial membrane potential, the rhodamine 123 assay was performed. In this assay, the lipophilic cationic dye rhodamine 123 was used to measure the mitochondrial function in the treated cells. Loss of membrane integrity and membrane potential (∆Ψ) in mitochondria of a cell are well known characteristic features of apoptosis [24]. When the membrane potential in the mitochondria decreases, uptake of rhodamine 123 by the affected cells also decreases, which ultimately results in an exponential decrease in the fluorescence signal. Results of the present study revealed that the untreated cells displayed a strong fluorescence intensity indicating their unaffected health with good growth and cell proliferation (Figure 5B). On the other hand, the fluorescence signal decreased remarkably in the cells treated with 10 μM β-caryophyllene (Figure 5C,D), which suggests a reduced membrane potential due to the disrupted mitochondrial membrane. In addition, a time-dependent apoptotic effect of β-caryophyllene was recorded as the intensity of fluorescence signal dropped upon duration of the treatment (Figure 5C,D). A similar effect can be seen in the cells treated with the standard drug 5-flourouracil (Figure 5E). The apoptotic index estimated for β-caryophyllene treatment on HCT 116 cells after 24 h treatment was 64 ± 0.04 (Figure 5F). The results can be compared in Figure 5F with those of the standard reference 5-fluorouracil. The results provide additional information about the apoptotic properties of β-caryophyllene, and suggest that the apoptosis induced by β-caryophyllene in HCT 116 cells occurs via both the DNA and mitochondrial pathways.

### 2.7. β-Caryophyllene Induces Chromatin Condensation and DNA Fragmentation in HCT 116 Cells

**Figure 6:**
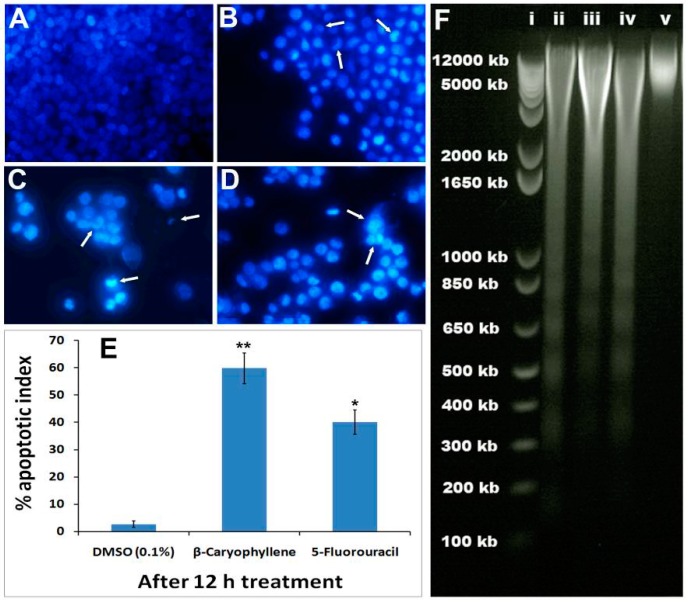
Photomicrographs depicting images of HCT 116 cells with Hoechst 33258 staining (**A**) Cells treated with vehicle (0.1% DMSO). The vehicle-treated cells revealed an intact cell membrane with an evenly distributed nucleus in cytosol; (**B**) Cells after 6 h of β-caryophyllene (10 µM) treatment. Cells treated with β-caryophyllene displayed early stage apoptotic symptoms such as membrane blebbing and chromatin condensation (arrows); (**C**) Cells after 12 h of β-caryophyllene (10 µM) treatment. The arrows indicate the advanced staged apoptotic signs such as of nuclear dissolution including the half-moon (crescent) shaped apoptotic nuclei. In addition, at several places, the arrows mark chromatin breakdown and fragmentation; (**D**) Cells treated with standard reference, 5-flourouracil (10 µM) also exhibited significant induction of apoptosis in the cells; (**E**) Graphical representation of percentage of apoptotic indices for HCT 116 and PANC-1 cells. The apoptotic index for each test group was expressed as a percentage of the ratio of number of apoptotic cells to the total number of cell in 10 different microscopic fields. Values are presented as mean ± SD (*n* = 10), * represents *p* < 0.05 and ** represents *p* < 0.01; (**F**) Effect of β-caryophyllene (10 µM) on DNA fragmentation in HCT 116 cells after 24 h treatment. (i). The standard DNA ladder; (ii). The DNA fragmentation pattern of HCT 116 cells treated with 5-flourouracil (10 µM); (iii). DNA fragmentation pattern of HCT 116 cells treated with β-caryophyllene (5 µM); (iv). DNA fragmentation pattern of HCT 116 cells treated with β-caryophyllene (10 µM); (v). DNA fragmentation pattern of HCT 116 cells treated with 0.1% DMSO (negative control).

HCT 116 cells were selected to study the effect of β-caryophyllene on nuclear changes and condensation using Hoechst 33342 stain. The vehicle-treated cells showed aggressively growing cells with prominent nuclei (Figure 6A), whereas, the characteristic signs of apoptosis were noticed in the morphology of the nuclei of the β-caryophyllene-treated cells. β-Caryophyllene affected the nuclear morphology in a time-dependent manner (Figure 6B,C). β-Caryophyllene at 10 μM concentration, caused significant nuclei condensation (arrows) after 6 h of treatment (Figure 6B). This was clearly evidenced by lumping and squeezing of nuclear material to give irregularly distributed chromatin in the cytosol of the cells, whereas, at a later stage of treatment (after 12 h) the nuclear material is converted to shrunken and crescent-shaped structures (arrows), indicating the advanced features of apoptosis (Figure 6C). At this stage, most of the treated cells showed discrete chromatin bodies suggesting the induction of karyorrhexis caused by β-caryophyllene. These results were compared with the standard drug, 5-fluorouracil (Figure 6D). β-Caryophyllene displayed more pronounced effects compared to the standard reference drug. The apoptotic indices (Figure 6E) for untreated HCT 116 cells were 1.7% ± 0.04%. However, following the treatment with β-caryophyllene for 24 h, the apoptotic indices against HCT 116 cells were significantly increased to 58.4% ± 7% (Figure 6E).

It is well studied phenomenon that apoptotic cells go through a series of morphological changes namely, membrane blebbing, chromatin condensation, nuclear membrance disruption, nuclear fragmentation and dissolution [25]. Recently, a study reported that β-caryophyllene induces apoptosis in a mouse blood cancer cell line through caspase-3 induction [26]. Similarly, another study reported that a derivative of β-caryophyllene, β-caryophyllene oxide, induces apoptosis through suppression of PI3K/AKT/mTOR/S6K1 pathways and ROS-mediated MAPKs activation [27]. In the present study, β-caryophyllene demonstrated clear signs of apoptosis in human colorectal carcinoma (HCT 116) cells.

In order to investigate the effect of β-caryophyllene on advanced stage of apoptosis, isolated DNA from β-caryophyllene-treated HCT 116 cells was analyzed by agarose gel electrophoresis. The result showed an obvious DNA fragmentation caused by β-caryophyllene as a dose-dependent laddering pattern was observed (Figure 6F). The findings of the present study coincide with those of Amiel *et al.* [26], who reported a DNA fragmentation effect of β-caryophyllene in a mouse lymphoma cell line. However, for the first time, the present study demonstrated induction of nuclear condensation and fragmentation by β-caryophyllene in human cancer cells. The study of Amiel [26] reported that β-caryophyllene activates the caspase-3 enzyme. Caspase-3 is an executioner enzyme in a caspase-dependent apoptosis cascade. Induction of caspase-3 activity in turn leads to chromatin condensation, degradation and dissolution. In the present study these facts were further confirmed and validated by Hoechst 33342 assay in β-caryophyllene-treated human colorectal cancer cells.

### 2.8. β-Caryophyllene Inhibits Motility and Invasion in HCT 116 Cells

**Figure 7:**
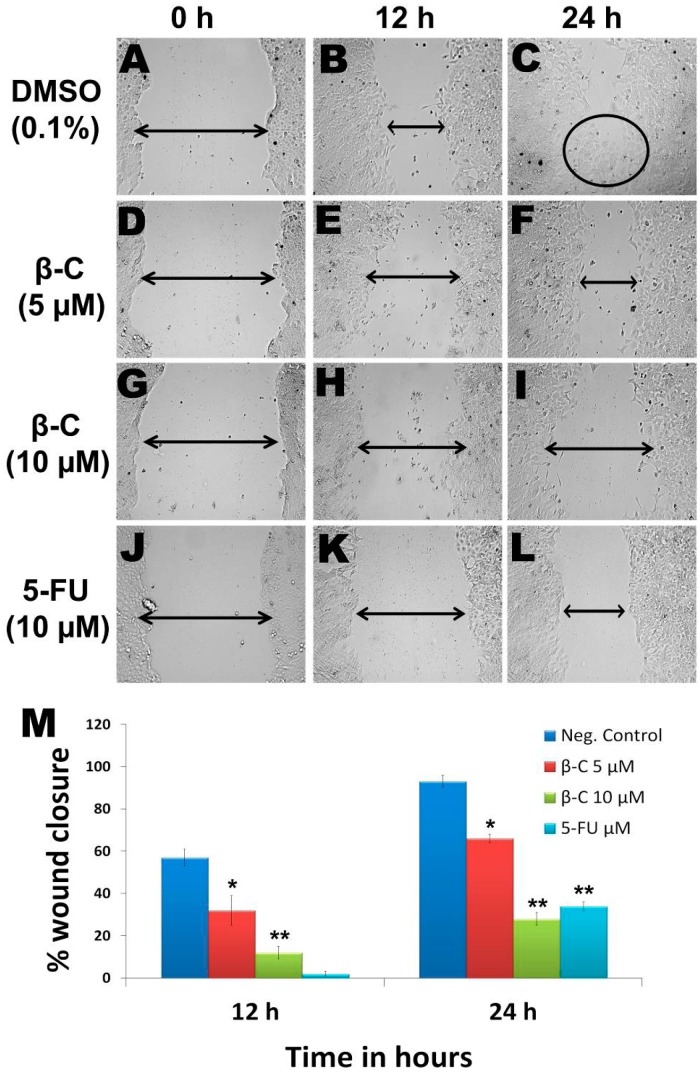
Due to the successful migration of HCT 116 cells in the untreated group (negative control), the wound is almost closed after 24 h (**A**–**C**), whereas in the β-caryophyllene-treated monolayer, the wound remained open even after 24 h incubation. β-Caryophyllene (5 μM) caused a significant inhibition of HCT 116 cell migration (**D**–**F**). Interestingly, even at a sub-cytotoxic concentration (10 μM), the compound caused significant inhibition of migration (**G**–**I**). The results can be compared with those of the standard reference 5-FU (**J**–**L**). Graphical representation (**M**) of the time and dose and time-dependent inhibitory effect of β-caryophyllene on migration of HCT 116 (values are in mean ± SD, *n* = 6, * *p* < 0.1, ** *p* < 0.005).

In the vehicle (0.1% DMSO)-treated group, the cells were successfully migrated after 24 h (Figure 7A,C), whereas β-caryophyllene exhibited a dose and time-dependent inhibitory effect on the motility of HCT 116 cells. The percentage of wound closure at sub-cytotoxic concentrations 5 (Figure 7D,F) and 10 μM (Figure 7G,I) was 66% ± 7% and 28% ± 2% inhibition, respectively at 24 h (*p* < 0.01). The inhibitory effect of β-caryophyllene was more pronounced compared to that of the standard reference, 5-fluorouracil (Figure 7J,L). At a concentration of 10 μM 5-fluorouracil produced 2 ± 1 and 34% ± 2% wound closure at 12 and 24 h, respectively (*p* < 0.05) (Figure 7M).

**Figure 8:**
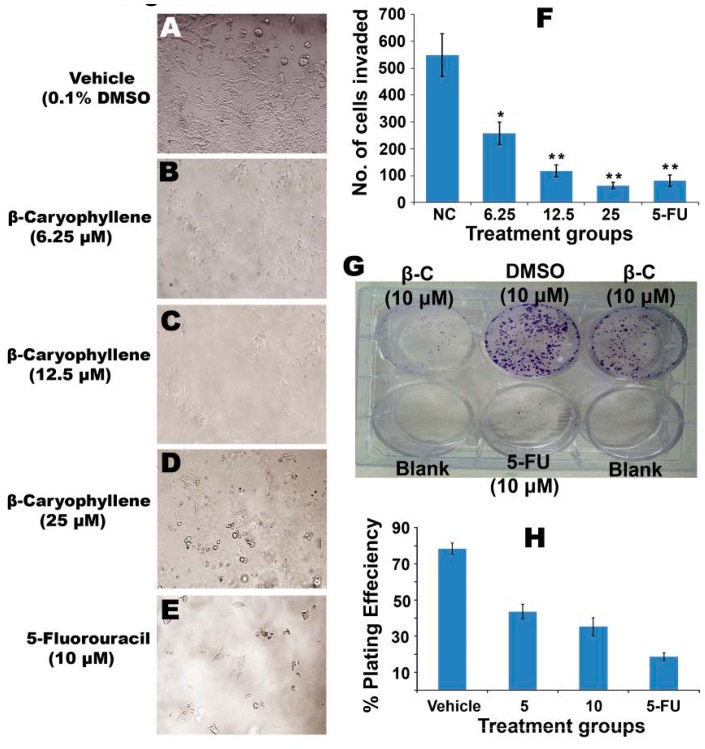
(**A**) Photomicrographs of HCT 116 cells invading the matrigel barrier. The negative control group showed a large number of invaded cells; (**B**‒**D**) Photomicrographs of HCT 116 cells showing the anti-invasion effect of β-caryophyllene (6.25, 12.5 and 25 µM, respectively) on a matrigel matrix; (**E**) Photomicrographic images of HCT 116 cells showing the anti-invasion effect of 5-flourouracil (10 µM) on a matrigel matrix; (**F**) Graphical representation of mean number of cells invaded per field of view, after counting 10 microscopic fields of view for triplicate wells. In untreated wells, the population of the cells invaded through the matrigel was significantly more than that of the treated wells (*p < 0.05, ** *p* < 0.01); (**G**) Effect of β-caryophyllene on survival of HCT 116 colonies in a colony formation assay. The picture clearly depicts the strong anti-clonogenic effect of β-caryophyllene on colonies of the cancer cells; (**H**) The graphical representation illustrates the percentage of plating efficiencies after the treatment of the cells with β-caryophyllene in comparison with negative control and the standard reference drug, 5-flourouracil. The results were presented as mean ± SD, *n* = 3.

Photomicrographs (Figure 8A) revealed the invasion of a large number of HCT 116 cells into the matrigel matrix, while β-caryophyllene (Figure 8B,D) caused significant (*p* < 0.05) inhibition. The total number of cells counted per microscopic field was 548 ± 79 for the negative control group. However, treatment with β-caryophyllene demonstrated significant (*p* < 0.001) obstruction of the cell invasion, as the total number of invaded cells in treatment group was found to be drastically lower than the untreated group. The effect of β-caryophyllene was compared with the positive control 5-flourouracil (Figure 8E). A dose dependent reduction was observed, as the concentrations 6.25, 12.5 and 25 μM, β-caryophyllene showed 258 ± 42, 118 ± 23 and 63 ± 12 invaded cells, respectively (Figure 8F). Induction of apoptosis in cancer cells controls various rate limiting factors that obstruct tumourigenesis and metastasis. Tumour cells easily detach from the tumour tissue and escape the primary site. The tumour cells enter the blood circulation, wherein they continue their proliferation and multiplication and localize into the secondary site. Metastasis occurs basically by the virtue of migratory and invasive cascades of the cancer cells. The ability of motility and invasion in cancerous cells is a prerequisite feature which promotes tumourigenesis and metastasis in body. Inhibition of *in vitro* migration of HCT 116 cells by β-caryophyllene indicates that it has a potential to suppress the motility of cancer cells and thus it could control metastatic propagation of malignancy [28]. Similarly, β-caryophyllene considerably restricted invasion of HCT 116 through the matrigel basement which strongly supports that notion that β-caryophyllene has an ability to interrupt cell invasion, a basic characteristic of metastatic cascade [29]. These findings support the anti-metastatic potential of β-caryophyllene against colorectal cancer.

### 2.9. β-Caryophyllene Inhibits Clonogenicity and the Growth of Tumor Spheroids

Colonization of cancer cells is one of the essential criteria for tumorigenesis. Figure 8G clearly shows the dose-dependent inhibitory effect of β-caryophyllene on colony formation of HCT 116 cells. The results showed significant suppression of colonization of the cancer cells. Percentage of plating efficiency (PE) in negative control (untreated cells) group was 78.4% ± 3%, which was drastically decreased upon treatment with β-caryophyllene. At concentrations of 5 and 10 µM, β-caryophyllene showed potent cytotoxic effects against the cancer cells as the percentage of plating efficiency recorded was 43.6% ± 4% and 35.2% ± 5%, respectively (Figure 8H). The graphical representation (Figure 8H) illustrates the quantitative estimation of the efficacy of β-caryophyllene against the clonogenicity of colorectal cancer cells. The results of colony formation assay suggested that β-caryophyllene can significantly stop colonization of HCT 116 cells.

**Figure 9:**
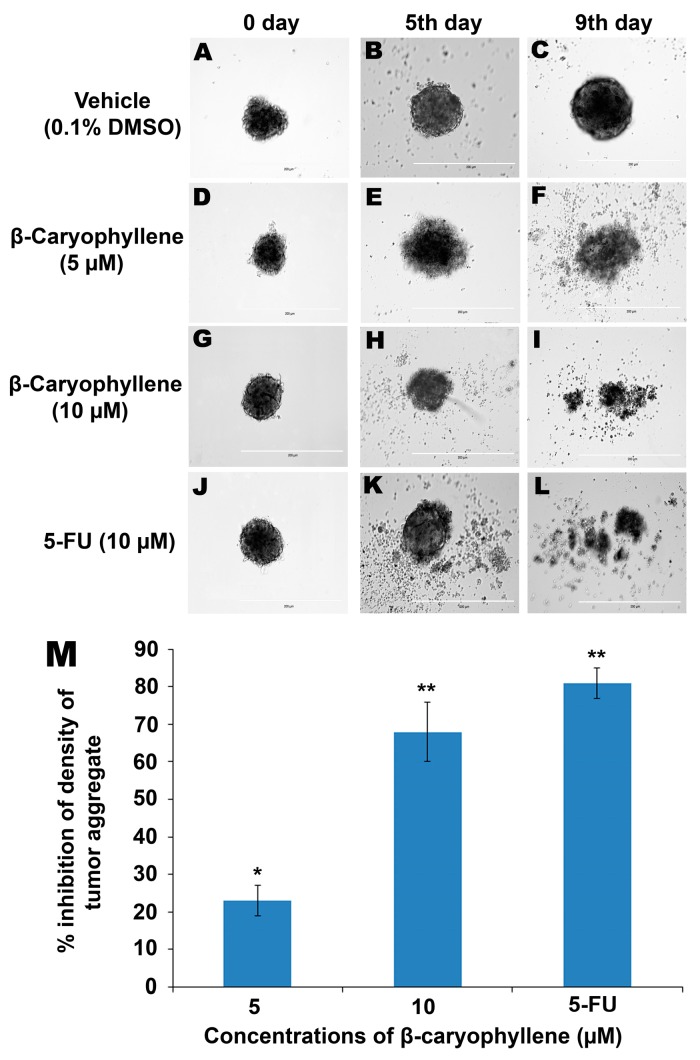
Anti-tumor aggregation effects of β-caryophyllene on *in vitro* HCT 116 cellular spheroids in a hanging drop assay. The cellular aggregates treated with vehicle (0.1% DMSO) developed in a solid spheroid shape within 9 days (**A**–**C**), whereas, β-caryophyllene at a concentration of 5 µM displayed significant inhibitory effect on the HCT 116 cellular aggregate microspheroids (**D**–**F**). At a concentration 10 µM, β-caryophyllene completely obliterated the solid cellular aggregates of HCT 116 cells on the 9th day of seeding (**G**–**I**). Similar effects were observed with the standard reference, 5-flourouracil (**J**–**L**). Graphical representation (**M**) of the dose dependent inhibitory effect of β-caryophyllene on *in vitro* cellular aggregates of HCT 116 cells on 9th day of treatment. The results are presented as mean ± SD, *n* = 6 (* *p* < 0.05, ** *p* < 0.01).

In a hanging drop environment, tumour cells aggregate and sediment in the cultures due to the applied gravitational force on the suspended cells. In the hanging drop assay, the cells were allowed to sediment and aggregate and later the cellular aggregates were harvested and seeded onto an agar-coated plate. The cellular aggregates from the hanging drop adopt a three-dimensional spheroid shape on the agar plate which leads to transformation of suspended cells into cellular aggregates which ultimately from a solid spheroid shape within the first 24 h (negative control, Figure 9A,C). Figure 9D,F illustrate the inhibitory effect of β-caryophyllene (5 µM) on the growth of aggregates and spheroids of HCT 116 cells. A stronger inhibitory effect of β-caryophyllene (10 µM) on tumour spheroids was recorded and is depicted in Figure 9G,I.

Initially, the cellular aggregates were prepared from 20 µL hanging drops (5000 cells/drop) which later on developed into a thick and dense tumor-shaped spheroid within 24 h as shown in the negative control group (Figure 9A,C). β-Caryophyllene displayed a dose-dependent inhibitory effect on the HCT 116 tumour spheroids. At concentrations of 5 and 10 µM, β-caryophyllene produced significant inhibitory effects of 23% ± 4% and 68% ± 8% on the density of the tumour spheroids, respectively (Figure 9D,I). The results are compared with that of the standard reference, 5-Fluorouracil which showed 81% ± 4% inhibition at 10 µM concentration (Figure 9J,L). Figure 9M illustrates the quantitative data of dose-dependent effect of β-caryophyllene in comparison with 5-fluorouracil. The inhibition of cellular aggregation is directly associated with the significant anti-proliferation effect on HCT 116 cells. The MTT assay in the present study supports the findings of the tumour spheroid assay.

## 3. Experimental Section

### 3.1. Chemicals and Reagents

Growth medium (RPMI 1640, Dulbecco’s Modified Eagle Medium, McCoy’s 5a modified medium and F-12K medium), trypsin and heat inactivated foetal bovine serum (HIFBS) were purchased from GIBCO (Paisley, UK). Methylthiazolyldiphenyltetrazolium bromide (MTT) reagent, phosphate buffer saline (PBS) and penicillin/streptomycin (PS) were purchased from Sigma-Aldrich (Darmstadt, Germany). Cell culture grade dimethyl sulfoxide (DMSO) was procured from Fluka (St. Louis, MO, USA). F-12K medium was obtained from ATCC (Rockville, MD, USA). All the reagents and solvents used were analytical grade. For biological assays, a stock solution (10 mM) of β-caryophyllene was prepared using DMSO. Further, various concentrations (3 to 100 µM) of β-caryophyllene were prepared by serially diluting the stock with respective culture medium.

### 3.2. Plants Material

Flowers, twigs and stem bark of *A. crassna* was collected from a local farm in Kajang, Selangor, Malaysia in 2013. Floral characteristics and taxonomical identification was confirmed by Mr. V. Shanmogan, a senior taxonomist from School of Biological Sciences, Universti Sains Malaysia. A herbarium sample (Ref. No. USM/122083) was submitted at the Department of Botany, School of Biological Sciences, UniversitiSains Malaysia, Pulau Pinang, Malaysia. In addition, the plant name has been checked and confirmed with The Plant List by the International Botanic Gardens [30].

### 3.3. Extraction of Essential Oils from A. crassna Bark

Fresh sample of stem bark of *A. crassna* was collected and rinsed with water. The covering layer of the bark composed of dead tissues was removed. The heartwood was sliced into small pieces and grinded mechanically. The powdered material of bark (500 g) was soaked in a round-bottom flask with 5 L of water at room temperature (25 ± 2 °C) for 5 days. Then, hydrodistillation was carried out at the boiling temperature of water for 48 h. The essential oils were collected for 12 h by a modified Clevenger-type apparatus, yielding a pale-yellow liquid (about 12.6 g).

### 3.4. Bioassay Guided Isolation of β-Caryophyllene

The obtained essential oil mixture of *A. crassna* was subjected to repeated chromatographic separation. Anti-proliferation (MTT) assay was used as the bioassay to guide the isolation of the active principle of the essential oil. The details of the chromatographic conditions and fractionation are given in the Supporting Information. The active compound was obtained as a brown-colored crystalline compound which was washed with *n*-hexane several times and recrystallized from hot methanol to obtain colourless β-caryophyllene (0.4 g, 0.2%).

### 3.5. Chemical Characterization Techniques

The structure of β-caryophyllene was elucidated using FT-IR and NMR spectral studies. Refer to the Supporting Information for detailed experimental conditions of the instruments (Figure S2).

### 3.6. Gas Chromatography-Mass (GC-MS) Spectral Analysis

Quantitative analysis of composition of the essential oils of *A. crassna* bark and the isolated compound, β-caryophyllene was carried out using GC-MS with the aim of comparingthe chemical profile of the crude extract and the pure compound. A detailed description of the assay conditions is given in the Supporting Information.

### 3.7. Antioxidant Activity of β-caryophyllene

#### 3.7.1. DPPH Radical Scavenging Activity

The antioxidant activity of β-caryophyllene was assessed based on the radical scavenging effect of stable 2,2-diphenyl-1-picrylhydrazyl (DPPH) free radical activity according to the method described previously [31].

#### 3.7.2. Ferric Reducing Antioxidant Power (FRAP) Assay

The FRAP assay was conducted according to the method described before [32]. The results were expressed as μM·Fe^2+^·mg^−1^. All measurements were carried out in triplicate and the mean values were calculated.

### 3.8. Microbial Strains

Six human pathogenic bacterial strains were procured from the Microbial Type Culture Collection Centre and Gene Bank (MTCC, Chandigarh, India). The strains used were *Bacillus cereus* (MTCC 1307), *Bacillus subtilis* (MTCC 6910), *Escherichia coli* (MTCC 732), *Klebsiella pneumonia* (MTCC 7028), *Staphylococcus aureus* (MTCC 7405) and *Pseudomonas aeruginosa* (MTCC 4302). Two fungal strains, *Rhizopus oryzae* (MTCC 1987) and *Tricho dermareesei* (MTCC 3929). All the bacterial strains were cultured on Mueller-Hinton agar medium at 37 °C and the fungal strains on Sabouraud dextrose agar medium at 28 °C.

### 3.9. Antimicrobial Assay

The antimicrobial activity of β-caryophyllene was determined by the disk diffusion method [33]. Refer to the Supporting information for the detailed methodology. Further, serial tube dilution technique [34,35] was used to determine MIC of β-caryophyllene.

### 3.10. Anticancer Assays

#### 3.10.1. Cell Lines and Culture Conditions

Panel of human cancer cells such as, pancreatic (PANC-1), colorectal (HCT-116 and HT-29), invasive squamous cell carcinoma (ME-180), leukemia (K562), hormone sensitive and invasive breast cancer cell line (MCF-7), and prostatic (PC3) adenocarcinoma cell lines were used. In addition, two normal cell lines, mouse fibroblast (NIH/3T3-L1), and human retinal ganglion cell line (RGC-5) were used as the model cell lines for normal cells. All the cell lines were purchased from ATCC. HCT 116, HT-29 and K562 cells were maintained in RPMI; MCF-7 cells were maintained in DMEM; ME-180 cells were maintained in McCoy’s 5a modified medium and PC3 was maintained in F-12K medium. All the media were additionally supplemented with 5% HIFBS and 1% PS. Cells were incubated in a humidified CO_2_ incubator at 37 °C supplied with 5% CO_2_.

#### 3.10.2. *In Vitro* Cytotoxic Assay

Inhibitory effect of β-caryophyllene on proliferation of the cell lines was tested using the MTT assay [36,37,38]. The selectivity index (SI) for the cytotoxicity of β-caryophyllene was calculated using the ratio of IC_50_ of the compound on a normal cell line (NIH-3T3) to the IC_50_ of the compound on cancer cell lines.

#### 3.10.3. Chromatin Condensation Assay using Hoechst 33342 Stain

Effect of β-caryophyllene on nuclear chromatin condensation in HCT 116 cells was assessed suing Hoechst 33258 staining [39] and quantified by fluorescence microscopy. Refer to the Supporting Information for the detailed methodology.

#### 3.10.4. DNA Fragmentation Assay

The assay was performed according to the method as described previously [39], with minor modifications. Refer to the Supporting Information for the detailed methodology.

#### 3.10.5. Rhodamin 123 Stain Assay

The effect of β-caryophyllene on mitochondrial membrane potential in treated-HCT 116 cells was studied and quantified by a rhodamine 123 assay [40]. Refer to the Supporting Information for the detailed methodology.

#### 3.10.6. Anti-Migration (Motility) Assay

The effect of β-caryophyllene on cellular migration (motility) was assessed on HCT 116 cells [41]. Refer to the Supporting Information for the detailed methodology.

#### 3.10.7. Cell Invasion

The effect of β-caryophyllene on HCT 116 cellular invasion was assessed using matrigel as an artificial basement membrane matrix following a previously described method [42]. Refer to the Supporting Information for the detailed methodology.

#### 3.10.8. Spheroid-Based *in Vitro* Anti-Tumor Assay

The effect of β-caryophyllene was tested on micro-spheroids (hanging drops) of HCT 116 cells. The micro-spheroids in this assay represents *in vitro* tumor and the assay was performed according to the previously described method [43]. Refer to the Supporting Information for the detailed methodology.

#### 3.10.9. Colony Formation Assay

The effect of β-caryophyllene on the clonogenicity of HCT 116 cells was investigated by a colony formation assay [42]. Refer to the Supporting Information for the detailed methodology.

### 3.11. Statistical Analysis

Statistical analysis was performed by one-way analysis of variance (ANOVA) followed by Tukey’s multiple comparison test with the help of IBM SPSS software (Version 20). Statistical significance were considered at *p* < 0.05 and *p* < 0.01 and were indicated as * and **, respectively.

## 4. Conclusions

The findings of the present work provide good evidence for β-caryophyllene being an active principle of *A. crassna* essential oil. The present study concludes that in particular β-caryophyllene could be a potential source of selective antifungal agents. Principally, from the findings of the present study, it can be concluded that β-caryophyllene has strong selective cytotoxic properties against human colorectal cancer cells. In addition, the results proved that the cytotoxicity induced by β-caryophyllene can be attributed to its apoptotic properties via DNA fragmentation and mitochondrial pathways. Further studies indicate that β-caryophyllene has promising capability to suppress tumour motility, cell invasion and tumour aggregation.

## Supplementary Materials

Supplementary materials can be accessed at: http://www.mdpi.com/1420-3049/20/07/11808/s1.
