# Supplementary material for: The Anticancer, Antioxidant and Antimicrobial Properties of the Sesquiterpene β-Caryophyllene from the Essential Oil of Aquilaria crassna
Source: Molecules. 2015 Jun 26;20(7):11808–29. doi: 10.3390/molecules200711808 (PMC6331975; doi:10.3390/molecules200711808)
Supplement: Supplementary file 1 [file molecules-20-11808-s001.pdf]

Supplementary Information

1. Method

1.1. Isolation of β-Caryophyllene using Silica Gel Column Chromatography

Approximately 300 g of heat-activated silica gel (0.04–0.06 mm, 60–120 mesh size) was mixed with n-hexane (500 mL) to make a silica gel slurry that was then packed into a (10 × 7 cm) column. About 5 gm of the essential oil mixture was loaded onto the column and a stepwise gradient elution was carried out starting with 100% dichloromethane. In the subsequent solvent systems, the polarity was gradually increased using methanol. The ratios of dichloromethane and methanol used were 9:1, 7:3, 1:1, 3:7 and 1:9, respectively. The final solvent system used was 100% methanol. The volume for each solvent system used was 25 mL. About 10 mL of eluents were collected throughout the process of elution. All the collected eluents were monitored by thin layer chromatography (TLC) plate. The eluents with similar Rf values were pooled together to obtain totally twelve fractions and the organic solvent was evaporated. Bioassay results indicated that out of 12 fractions, fraction 8 (0.9 g), which displayed the most pronounced anti-proliferation activity, was further applied to a column (30 × 2 cm) packed with silica gel (65 g) of particle size 0.063–0.200 mm. A step-wise gradient elution was carried out with a solvent system started with 100% *n*-hexane followed by increasing polarity from n-hexane to ethyl acetate and then to methanol. The proportion of *n*-hexane used with both solvents (ethyl acetate and methanol) was 75, 50, 25% and the volume of each solvent system used was 25 mL. The eluents (5 mL each) were collected from the column and continuously monitored using TLC. Eluents with similar R_f values were combined together to obtain three fractions. Fraction 3 yielded a brown colored crystalline compound which was washed with n-hexane several times and recrystallized with hot methanol to obtain colourless β-caryophyllene (0.4 g). The structure of β-caryophyllene was elucidated using FTIR, ^{1}H- and ^{13}C-NMR and GC-MS spectral studies.

1.2. Characterization of β-Caryophyllene

All the solvents used during extration were of reagent grade and no imputies, due to solvent, were found in the isolated β-caryophyllene during characterization. FT-IR was performed using a Perkin Elmer 2000FT-IR instrument (Perkin Elmer, Winter St., Waltham, MA, USA). Since the isolated compound was a liquid the thin layer method was used to collect the spectral features. Accordingly, the compound was layered between the thallium bromide discs and exposed to IR irradiations. NMR (^{1}H and ^{13}C) analysis was performed in deutrated chloroform ($CDCl_3$) at 500 MHz for ^{1}H and 125 MHz for ^{13}C nucli on a 500 MHz NMR machine ((Bruker 500 MHz, Vernon Hills, IL, USA). The molecular weight of the compound was determined by gas chromatography-mass spectrometry (GC-MS: 6890N/5973 Agilent Technologies-Hewlett Packard Model (Santa Clara, CA, USA). The sample was prepared in HPLC grade methanol (10 mg·mL^{-1}) and was filtered through 0.45 micron filter.

1.3. Characteristic Features of FT-IR and ^{1}H-NMR spectra

(FT-IR, ν·cm^{-1}): 2948, 2948, 2858 ($C_{sp}{}^3$-H, CH_3- and -CH_2- stretching), 1633 (C=C non-aromatic stretching) 1446, 1389 (-CH_2-, bending). ^{1}H-NMR ($CDCl_3$, δ ppm): 5.29–5.37 (1H, m), 4.87 (1H, s), 4.99 (1H, s), 1.93–2.56 (6H, m), 1.47–1.76 (6H, m), 1.03 (3H, s), 1.05 (3H, s).^{13}C{^{1}H} NMR ($CDCl_3$,

δ ppm): 154.5, 135.3, 124.5, 111.7, 53.5, 48.5, 40.5, 34.8, 30.1, 29.4, 28.4, 22.6, 16.3. GC-MS (624 scans, 7.38 min) *m*/*z* 204.

1.4. Gas Chromatography-Mass (GC-MS) Spectral Analysis

Chemical composition of extract, fractions and sub-fractions was studied using the fragmentation pattern in mass spectrum of GC-MS. The analysis was conducted with the help of the Metabolites Spectral Database and NIST (National Institute of Standards and Technology, Gaithersburg, MD, USA) Library. The major chemical components were identified on the basis of similarity index (SI), Wiley 8 computer library. The analysis was done by comparing the mass spectrum of unknown compounds compared with the known compounds stored in the library. The parameters such as, retention time, nomenclature, molecular weight, structure, and composition of the major components were noted down (Table S1). The assay conditions were as follows: HP-5MS capillary column (30 m × 0.25 mm ID × 0.25 µm, film thickness); held at 70 °C for 2 min, raised to 285 °C at a rate of 20 °C/minute and held for 20 min; 285 °C for MSD transfer line heater; carrier helium at a flow rate of 1.2 mL/min; 2:1 split ratio. About 1 µL solution of SF-1 in chloroform (10 mg·mL^{-1}) was injected automatically. Scan parameter low mass: 35 and higher mass: 550. The constituents were identified by comparison with standards using NIST 02. A total ion chromatogram (TIC) was used to compute the percentage of the identified constitutes. Results of the GC-MS analysis with retention time (R_t), % area peak, molecular formula and molecular weight for all the major chemical components present in the essential oils of *Aquilaria crassna* are given in the Table S1. The mass fragmentation for the major chemical constituents identified is given in the Figure S1A–M

Table S1: GC-MS quantitative estimation of phytochemicals of *A. crassna* essential oils

Peak	R_t (min)	Area %	Phytoconstituents	Molecular Formula	Molecular Weight	Calculated (m^{+1} Ionic Peak) [a]
a	7.26	3.925	Octahydro-tetramethyl-Cycloprop[*e*]azulene	$C_{15}H_{24}$	204	205
b	7.38	8.111	β-Caryophyllene	$C_{15}H_{24}$	204	205
c	7.47	2.694	Octahydro-tetramethyl-Cyclopropa[*a*]naphthalene	$C_{15}H_{24}$	204	205
d	7.64	4.755	α-Caryophyllene	$C_{15}H_{24}$	204	205
e	8.78	1.72	Caryophyllene oxide	$C_{15}H_{24}O$	220	220
f	8.96	6.193	2-Naphthalenemethanol	$C_{15}H_{26}O$	222	223
g	9.68	1.866	Methyl-phenyl-pyrrolidinedione	$C_{11}H_{11}NO_2$	189	190
h	9.98	1.377	Diallyl-cyclohexanone	$C_{12}H_{18}O$	178	179
i	11.83	1.924	Isobornyl propionate	$C_{13}H_{22}O$	210	211
j	12.16	1.685	9-H-Cycloisolongifolene	$C_{15}H_{22}O$	218	219
k	12.26	2.583	3-Bromo-Cyclodecene	$C_{10}H_{17}Br$	218	219
l	12.46	7.103	1-Phenanthrenecarboxylic acid	$C_{16}H_{22}O_4$	318	319
m	13.01	4.642	Benzenedicarboxylic acid	$C_{16}H_{22}O_4$	278	279

[a] NIST Mass Spec Data Center, S.E. Stein, director.

1.5. Antimicrobial Assay

A suspension (0.1 mL) of the test microorganism (1 × 10^8 cells·mL^{-1}) was spread on Mueller-Hinton agar plates for bacteria and Sabouraud Dextrose Agar forthe fungi. Sterile 6 mm disks, impregnated

with various concentrations (3.25–100 μM) of β-caryophyllene were placed on the microbial lawns. The bacterial plates were incubated at 37 °C for 24 h, whereas the fungal plates were incubated at 30 °C for 48 h. DMSO (0.1%) and kanamycin (25 μM) were used as negative and positive controls, respectively. Microdilution technique was employed to estimate minimum inhibitory concentration (MIC) and the results are presented as mean ± S.D. Triplicate tests were carried out in all experiments.

1.6. Determination of Nuclear Condensation by Hoechst 33342 Stain

PANC-1 and HCT 116 cells were treated with β-caryophyllene (10 μM) and analyzed separately at two different time intervals (6 and 12 h). DMSO (0.1%) and 5-fluorouracil (10 μM) were used as negative and positive controls, respectively. The cells were fixed in 4% paraformaldehyde for 20 min before staining with Hoechst stain 33342 (1 $\mu g \cdot mL^{-1}$ in PBS) for 20 min. Nuclear morphology was examined under a fluorescent microscope (TECAN Multi-mode microplate reader Model Infinite 200 (Mannedorf, Switzerland)). Cells with bright colored, condensed or fragmented nuclei were considered as apoptotic. The number of cells with apoptotic morphology was counted in randomly selected fields per well. The cells were photographed at 20× magnification, using a digital microscope (Advanced Microscopy Group, Model: EVOS f1, Staley Rd. Grand Island, NY 14072, USA). The apoptotic index was calculated as a percentage of apoptotic nuclei compared to the total number of cells and presented as the mean ± SD (n = 8).

1.7. DNA Fragmentation Assay

HCT 116 cells (5×10^6) were seeded in 6-well plate and allowed overnight for attachment. Then the cells were treated with two different concentrations of β-caryophyllene for 24 h. Later, the cells were harvested, homogenized and DNA was extracted with Wizard® SV Genomic DNA Purification kit (Promega, Woods Hollow Road Madison, WI, USA). DNA was subjected to electrophoretic separation for 2 h at 100 V in 1.2% agarose gel stained with ethidium bromide. DNA fragmentation profile was observed under UV illumination and visualized using a gel documentation system (Bio-Rad Laboratories, Inc., Gel Doc EZ™ imager, Hercules, CA, USA). The separated DNA fragments were matched with the standard DNA ladder (Invitrogen™, Carlsbad, CA, USA).

1.8. Detection of Mitochondrial Membrane Potential by Rhodamine 123 Staining

HCT 116and PANC-1 cells were separately seeded in 6 well plates for overnight for attachment. The cells were treated with β-caryophyllene at 10 μM concentration, for 6 and 12 h intervals and then fixed using 4% paraformaldehyde for 20 min. DMSO (0.1%) and 5-fluorouracil (10 μM) were used as negative and positive controls, respectively. Rhodamine 123 was added to cells at a final concentration of 5 μg/mL and incubated for 30 min to stain the mitochondria. The wells then were photographed using inverted EVOS f1 digital microscope at 20× magnification power to monitor the fluorescent signals.

1.9. Cell Migration Assay

HCT 116and PANC-1 cells were seeded separately in 6-well plate and incubated for 48 h to achieve almost 100% confluent monolayer. A straight scratch was created using a 200 μL micropipette tip, and

the cells were treated immediately with β-caryophyllene (20 and 40 μM), or 0.1 % DMSO. The wound was photographed at zero, 12 and 18 h. The distance of cell-free area was measured using the Leica Quin software, and the results are presented as average of percentage of inhibition of migration in comparison to the negative control (± SD, n = 6).% inhibition of cell migration = [1 − (the width at the indicated times/the width at zero time)] × 100.

1.10. Cell Invasion

In this assay, matrigel in growth medium (1:1) was taken in 6-well plate and incubated for 45 min to solidify. Each cell line (5×10^3 $well^{-1}$) was seeded separately into the matrigel coated wells, and treated for 24 h with 0.1% DMSO or β-caryophyllene (concentrations, 20 and 40 μM). Subsequently, the upper media was carefully aspirated and non-invading cells were washed off gently, and cells were photographed microscopically. Invaded cells were counted and the result is reported as percentage inhibition of invasion in treated cells relative to untreated cells.

1.11. Hanging Drop Spheroid Assay

PANC-1 and HCT 116 cells (2.5×10^5 cells/mL) were cultured in the growth media. The confluent cultures were trypsinized, washed with phosphate buffered saline, and resuspended in the media. Drops (20 μL) of medium containing 5000 cells/drop were placed onto the lids of 100 mm dishes, which were inverted over dishes containing 10 mL the growth medium to maintain humidity. Hanging drop cultures were incubated overnight for sedimentation. The resulting cellular aggregates (spheroids) were harvested using a Pasteur pipette under a dissecting microscope and introduced into a 100 mm dish base-coated with 0.75% agar and filled with 10 mL the growth media. Three series of 25 drops were prepared for each trial, and the hanging drop sedimentation time, time on agar, as well as cell concentration per drop were assessed.

1.12. Colony Formation Assay

HCT 116 cells (500 cells·mL^{-1}) were separately seeded in 6-well plate and incubated for 12 h. Subsequently, the cells were treated for 48 h with β-caryophyllene (10, 20, 40 and 60 μM), or 5-fluorouracil (10 μM) or 0.1% DMSO. The cells were maintained until sufficiently large colonies (≥50 cells) were produced for 10 days. The colonies were fixed, stained with 0.2% crystal violet and counted under stereomicroscope. Percentage of plating efficiency (PE %) and percentage of surviving fraction (SF %) was calculated. The results are presented as the mean ± SD (n = s 3).

2. Characterization of β-Caryophyllene Using FT-IR and NMR Spectral Studies

2.1. FT-IR Spectroscopy

The compound was isolated as a light yellow to colourless oily product. FT-IR spectral features of purified compound showed three distinct vibrational bands (2948, 2925 and 2858 cm^{-1}) representing the presence of alkyl groups (-CH_2- and -CH_3) (Iqbal *et al.*, 2013 [1]). Furthermore, appearance of a vibrational band at 1633 cm^{-1} indicated the presence of C=C group(s) in the molecule (Chauhan *et al.*,

2013 [2]; Goksel *et al.*, 2013 [3]; İbis *et al.*, 2013 [4]). The C=C could either be aromatic or non-aromatic, since the stretching vibrations in the range 300–3100 $cm^{-1}$for C_{sp2}-H aromatic ring stretch were not observed, hence the indicated C=C vibrations were classifiedas non-aromatic. The other vibrations at 1446, 1389, 1367 cm^{-1} appeared for the -CH_2- bendings.

2.2. NMR Spectroscopy

The isolated compound was further analyzed by ^{1}H- and ^{13}C-NMRspectroscopic technique. ^{13}C-NMR spectrum showed signals ranging δ 16.3–40.5 ppm for the methyl and in ring methylene groups (indicated by A, Figure S2a). Furthermore, the cyclobutane and cyclononene adjoining carbons, 5 and 6, appeared at 53.7 and 48.5 δ ppm, respectively. The terminal carbon of non-cyclic methylene group 4 appered at 111.7 whereas related methylene carbon 1 apperead in the most downfield region at 154.6 δ ppm. The cyclononene methylene carbons 2 and 3 appeared at 135.1 and 124.6 δ ppm, respectivley.

Similarly, ^{1}H-NMR spectrum indicated a most downfield multiplet signal ca. at 5.32 δ ppm for the cyclononene alkene proton 1 (see Figure S2b). Terminal ethylene protons, indicated by 2, appeared in the range 4.87–4.99 δ pm. The methyl group 3 of cyclononene appeared at about 1.65 whereas the methyl groups, indicated by 4, appeared in relatively upfield region at about 1.04 δ ppm. Before extending to the biological applications, all the characterisitic signals (^{13}C and ^{1}H) of the isolated compound were compared with the reported Caryophyllene derivatives (Gohari *et al.*, 2005 [5]; Sköld *et al.*, 2006 [6]) to verfy the title compound.

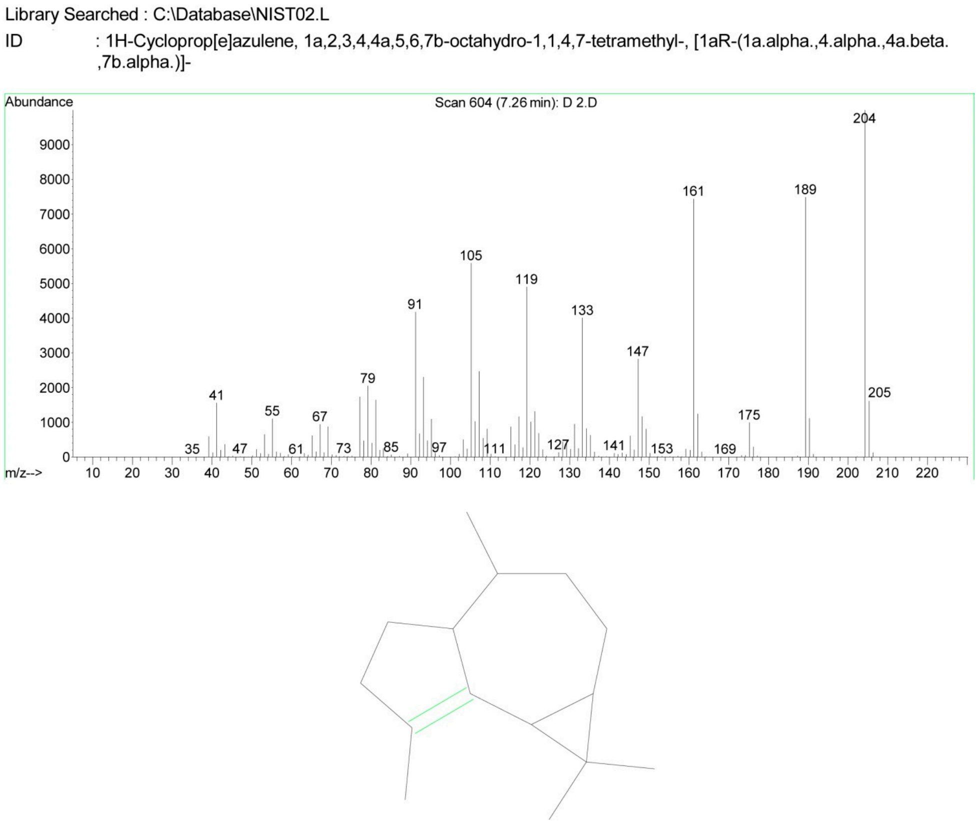

(A)

Figure S1. *Cont.*

Library Searched : C:\Database\NIST02.L
ID : Caryophyllene

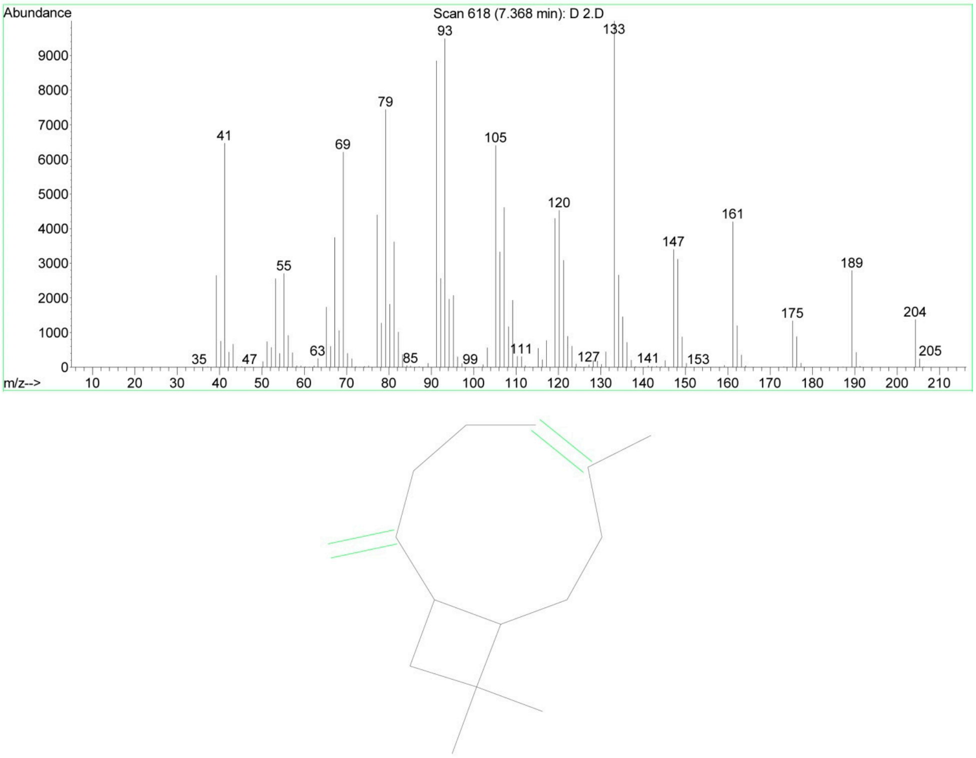

(**B**)

Library Searched : C:\Database\NIST02.L
ID : 1H-Cyclopropa[a]naphthalene, 1a,2,3,5,6,7,7a,7b-octahydro-1,1,7,7a-tetramethyl-, [1aR-(1a.alpha.,7.alpha.,7a .alpha.,7b.alpha.)]-

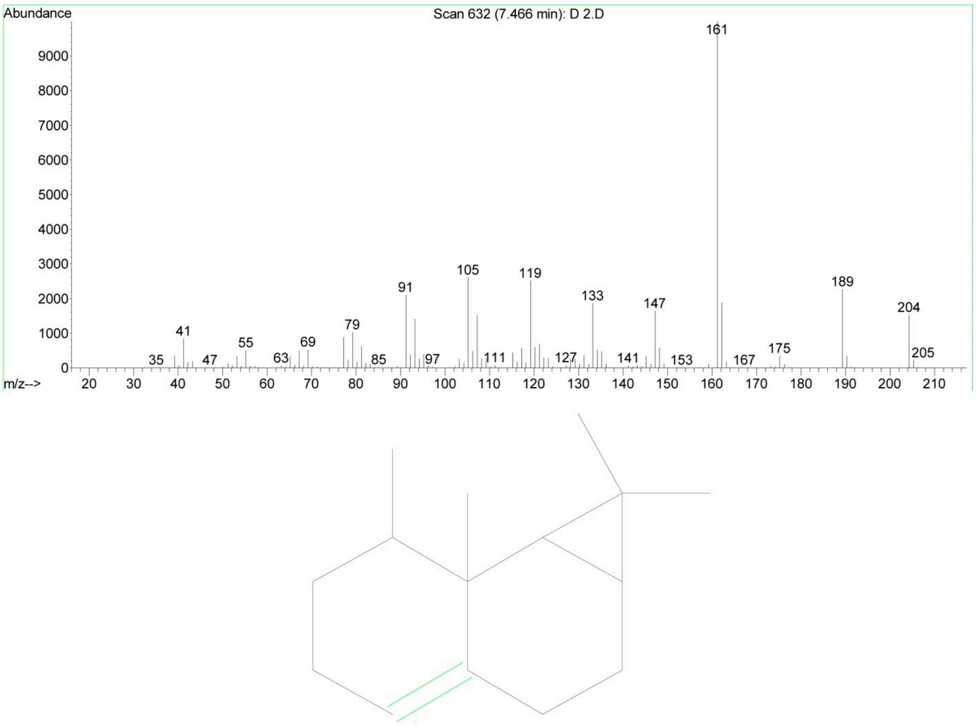

(**C**)

Figure S1. *Cont.*

Library Searched : C:\Database\NIST02.L
ID : .alpha.-Caryophyllene

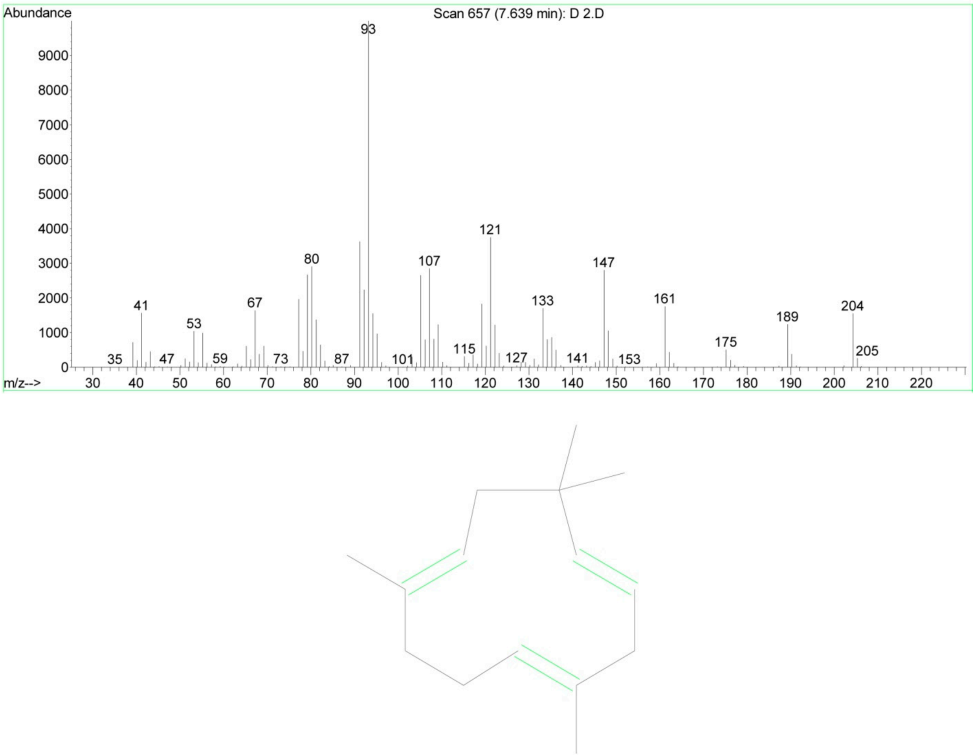

(**D**)

Library Searched : C:\Database\NIST02.L
ID : Caryophyllene oxide

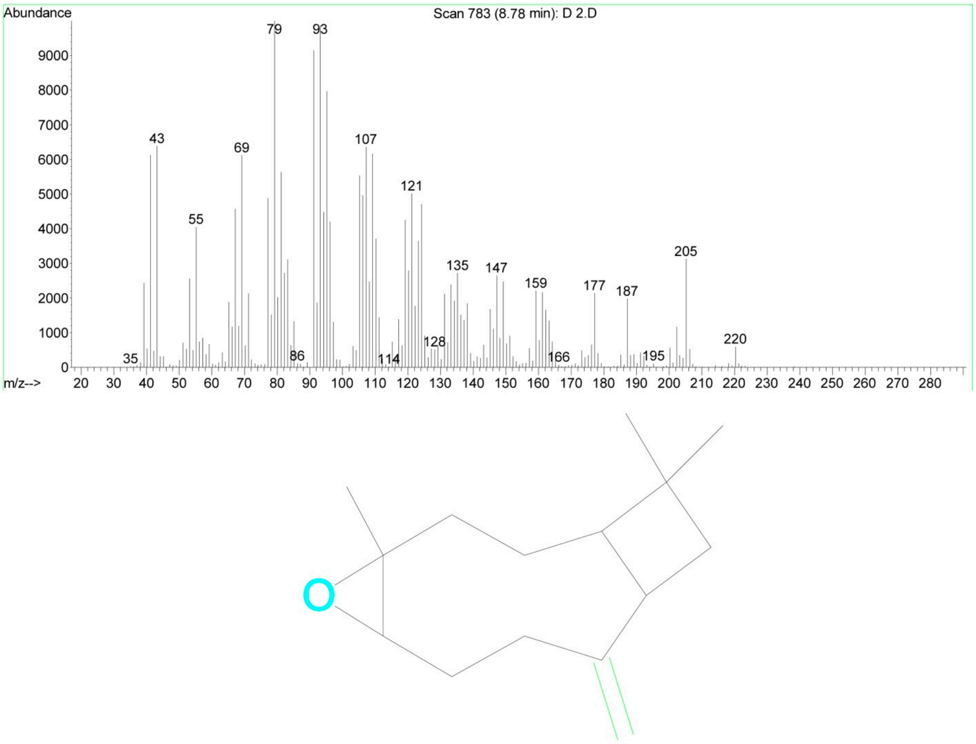

(**E**)

Figure S1. *Cont.*

Library Searched : C:\Database\NIST02.L
ID : 2-Naphthalenemethanol, decahydro-.alpha.,.alpha.,4a-trimethyl-8-methylene-, [2R-(2.alpha.,4a.alpha.,8a.beta.)]-

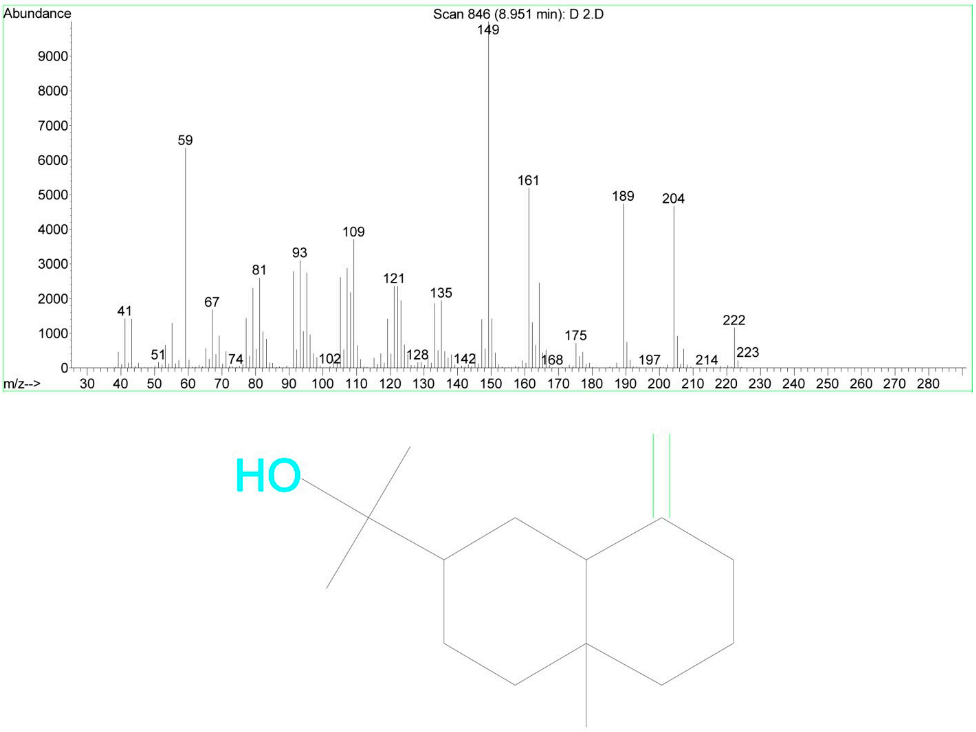

(**F**)

Library Searched : C:\Database\NIST02.L
ID : 2,5-Pyrrolidinedione, 3-methyl-1-phenyl-

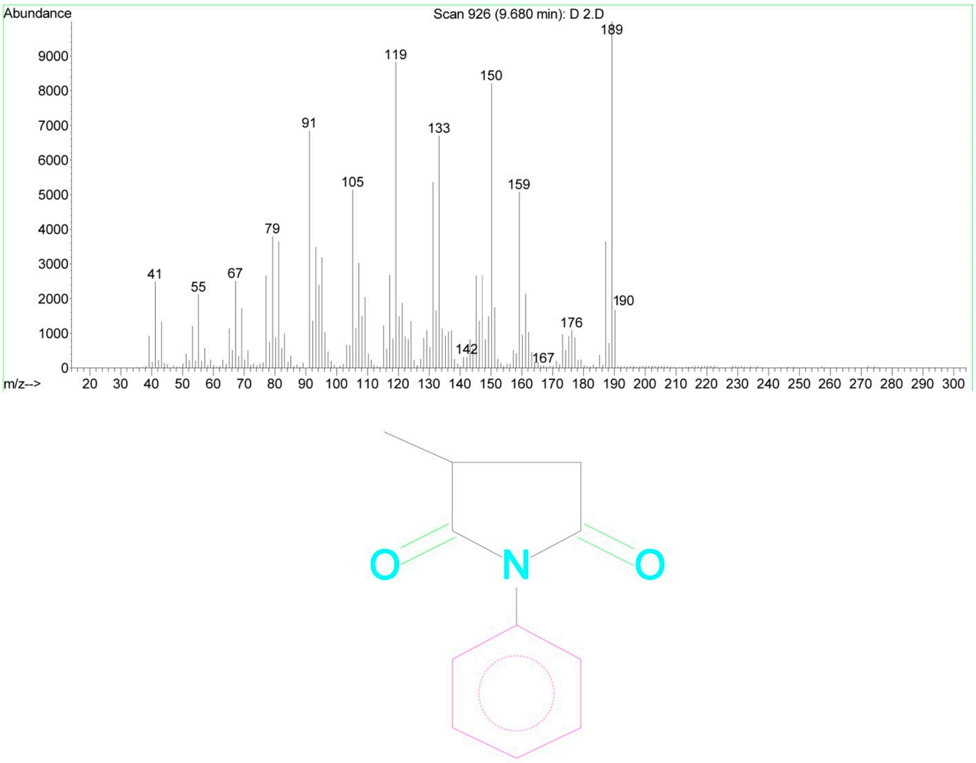

(**G**)

Figure S1. *Cont.*

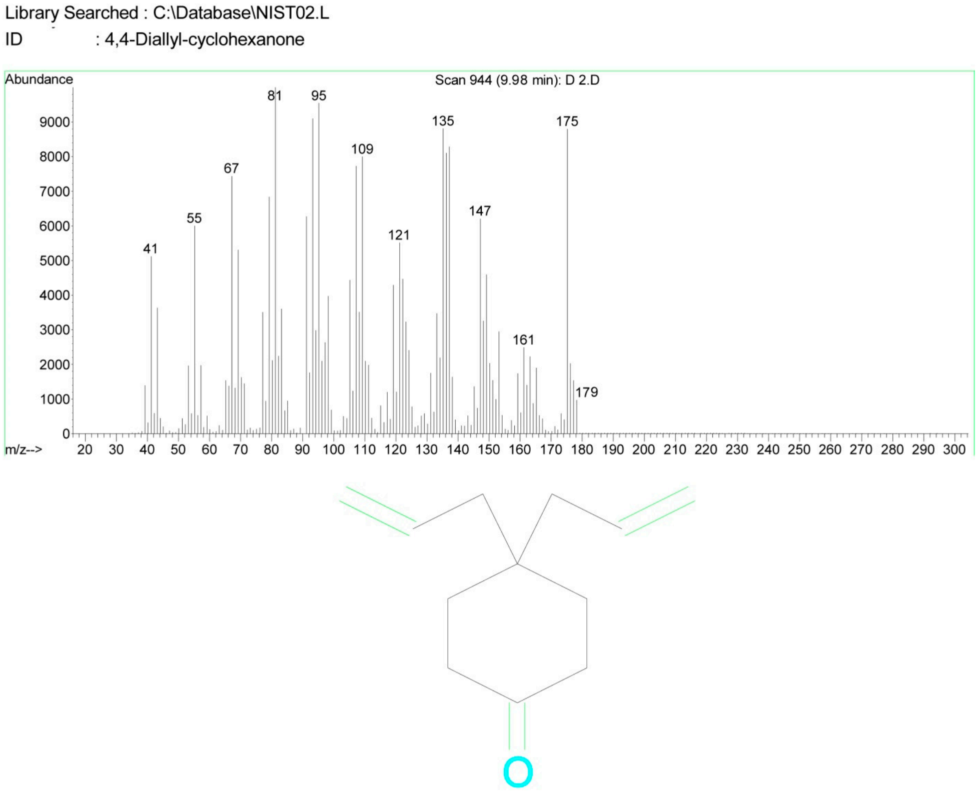

(H)

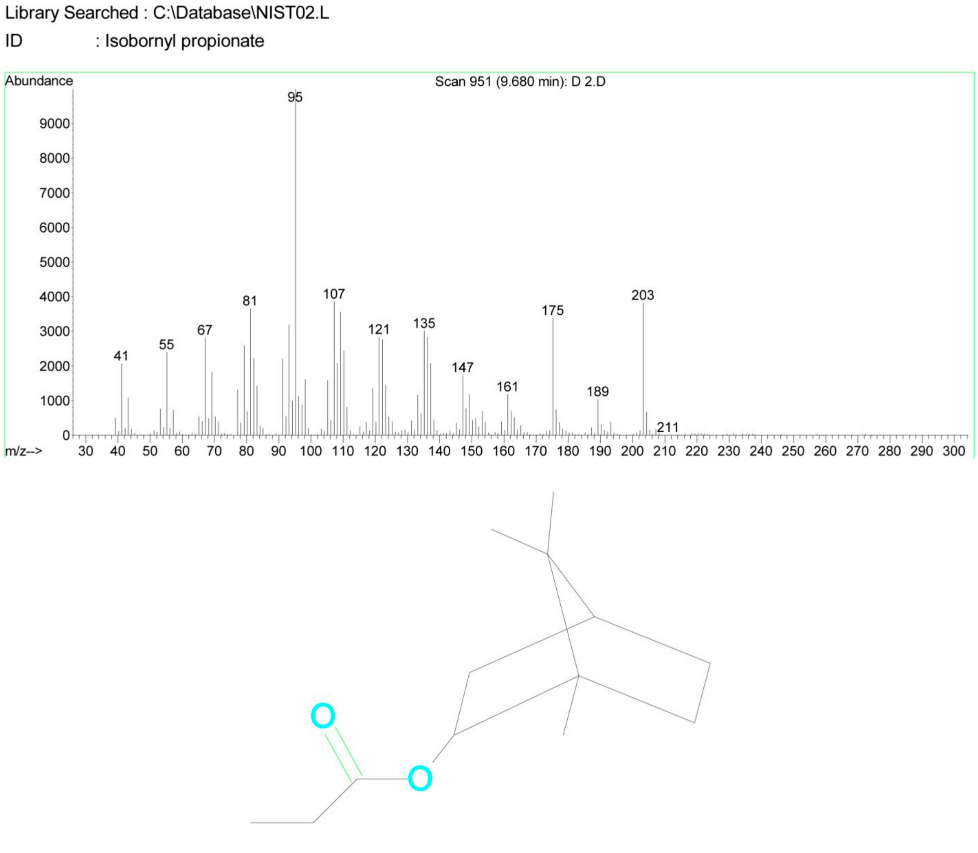

(I)

Figure S1. *Cont.*

Library Searched : C:\Database\NIST02.L
ID : 9H-Cycloisolongifolene, 8-oxo-

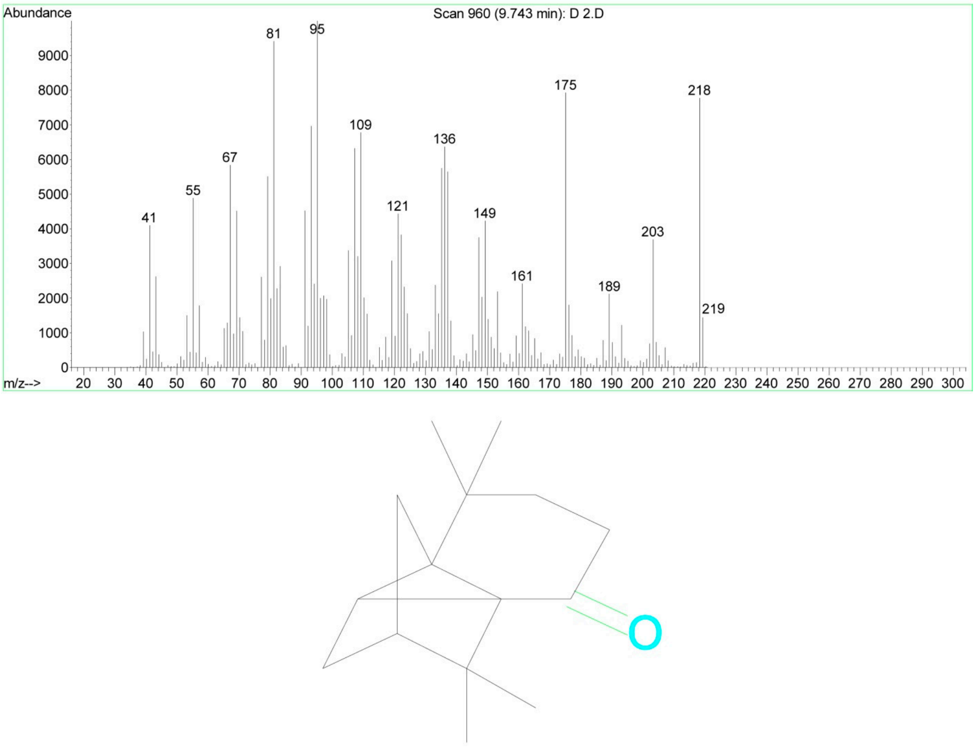

(J)

Library Searched : C:\Database\NIST02.L
ID : Cyclodecene, 3-bromo-

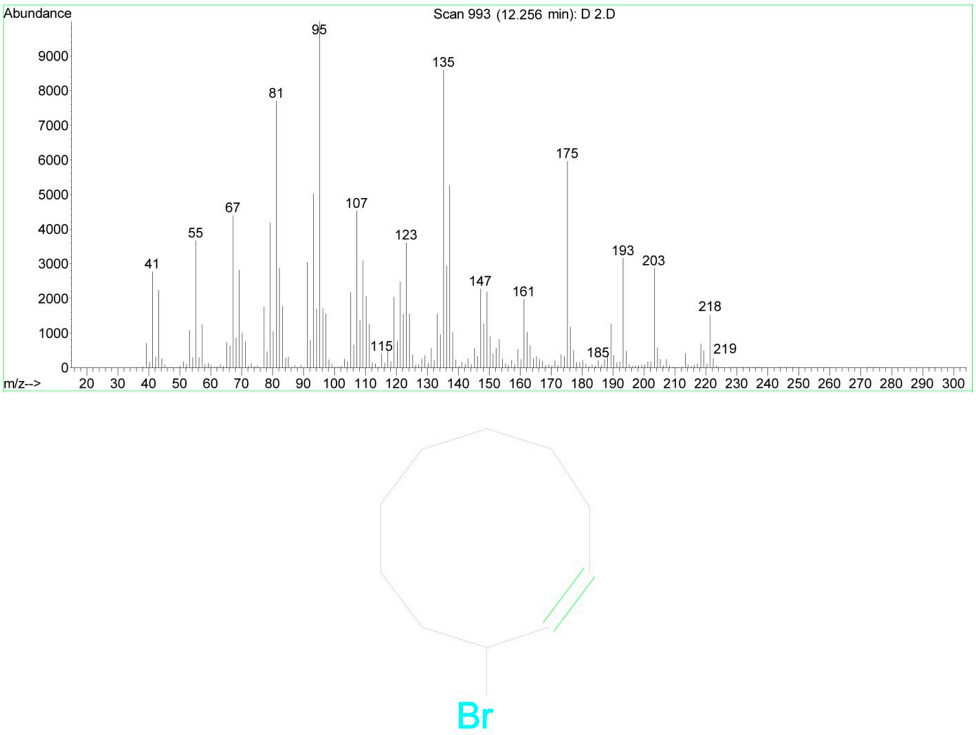

(K)

Figure S1. *Cont.*

Library Searched : C:\Database\NIST02.L

ID : 1-Phenanthrenecarboxylic acid, 1,2,3,4,4a,5,6,7,8,9,10,10a-dodecahydro-1,4a-dimethyl-7-(1-methylethyl)-, met hyl ester, [1R-(1.alpha.,4a.beta.,7.beta.,10a.alpha.)]-

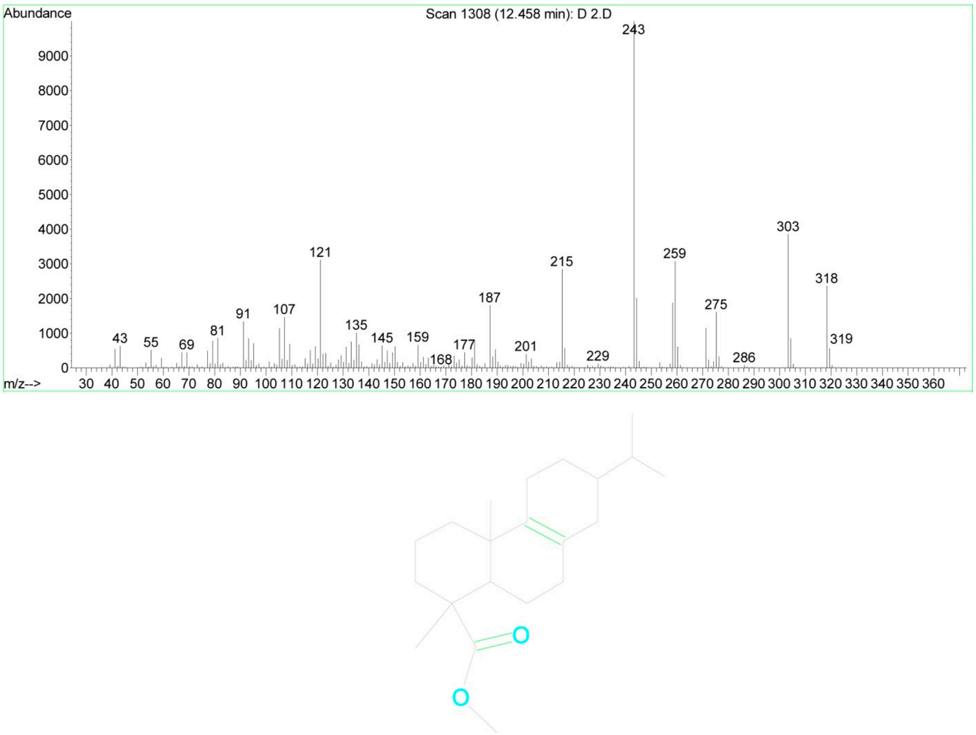

(**L**)

Library Searched : C:\Database\NIST02.L

ID : 1-Phenanthrenecarboxylic acid, 1,2,3,4,4a,5,6,7,8,9,10,10a-dodecahydro-1,4a-dimethyl-7-(1-methylethyl)-, met hyl ester, [1R-(1.alpha.,4a.beta.,7.beta.,10a.alpha.)]-

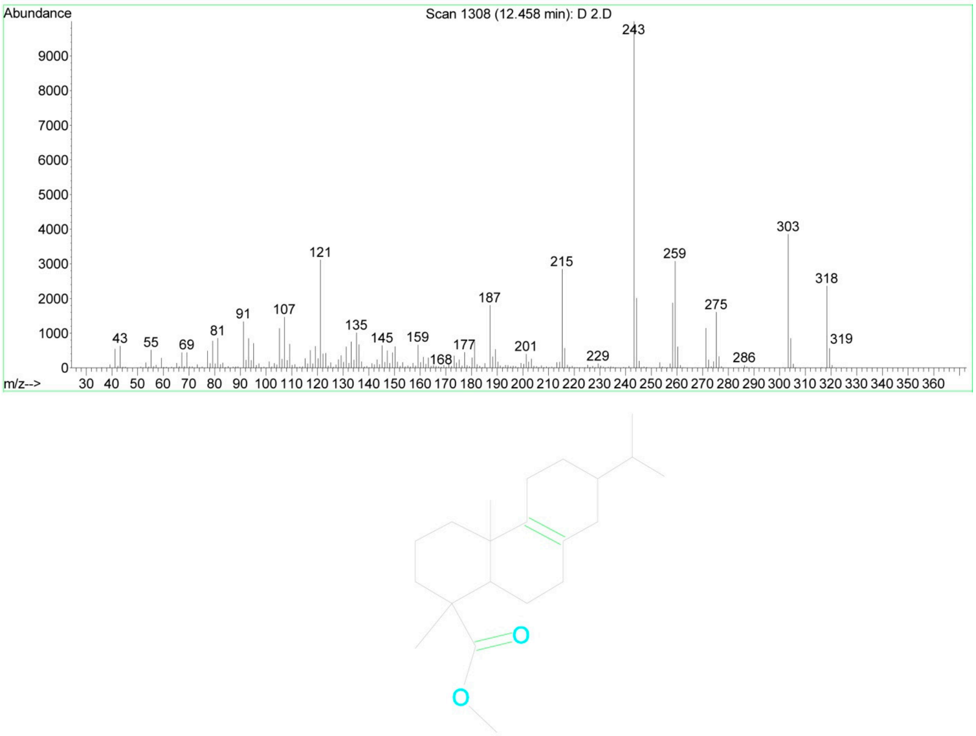

(**M**)

Figure S1. (**A**): Mass spectra for the chromatographic peak "a", showing the characteristic fragmentation pattern for Octahydro-tetramethyl-Cycloprop[e]azulene; (**B**): Mass spectra for the chromatographic peak "b", showing the characteristic fragmentation pattern for β-Caryophyllene. (**C**): Mass spectra for the chromatographic peak "c", showing the characteristic fragmentation pattern for Octahydro-tetramethyl-Cyclopropa[a]naphthalene; (**D**): Mass spectra for the chromatographic peak "d", showing the characteristic fragmentation pattern for α-Caryophyllene; (**E**): Mass spectra for the chromatographic peak "e", showing the characteristic fragmentation pattern for Caryophyllene oxide; (**F**): Mass spectra for the chromatographic peak "f", showing the characteristic fragmentation pattern for 2-Naphthalenemethanol; (**G**): Mass spectra for the chromatographic peak "g", showing the characteristic fragmentation pattern for Methyl-phenyl-pyrrolidinedione; (**H**): Mass spectra for the chromatographic peak "h", showing the characteristic fragmentation pattern for Diallyl-cyclohexanone; (**I**): Mass spectra for the chromatographic peak "i", showing the characteristic fragmentation pattern for Isobornyl propionate; (**J**): Mass spectra for the chromatographic peak "j", showing the characteristic fragmentation pattern for 9-*H*-Cycloisolongifolene; (**K**): Mass spectra for the chromatographic peak "k", showing the characteristic fragmentation pattern for 3-Bromo-Cyclodecene; (**L**): Mass spectra for the chromatographic peak "l", showing the characteristic fragmentation pattern for 1-Phenanthrenecarboxylic acid; (**M**): Mass spectra for the chromatographic peak "m", showing the characteristic fragmentation pattern for Benzenedicarboxylic acid.

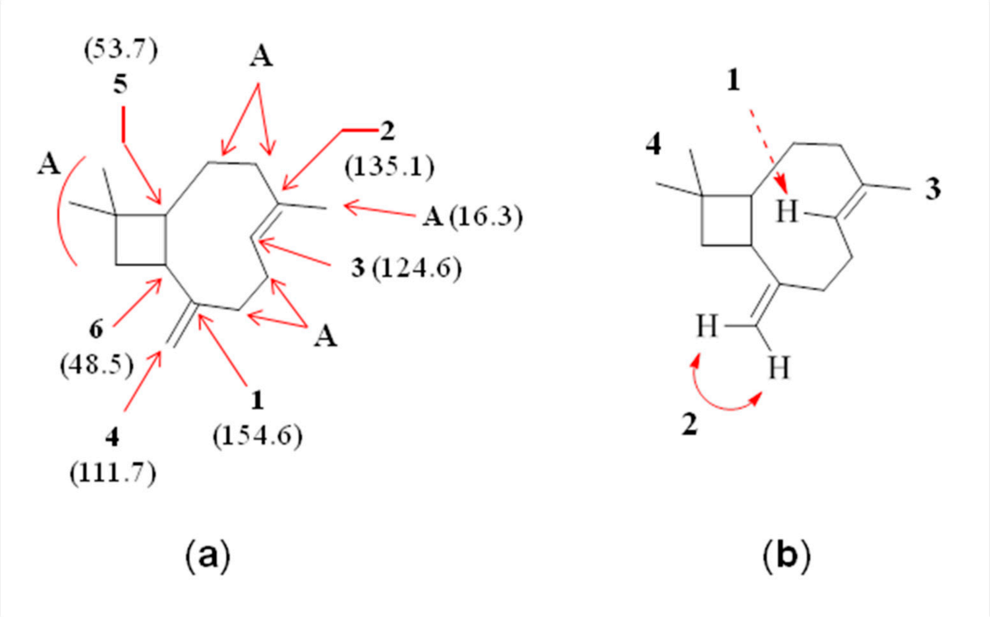

Figure S2. (**a**) Chemical structure of β-caryophyllene illustrating the ^{13}C assignments obtained from ^{13}C-NMR spectrum. (**b**) Chemical structure of β-caryophyllene depicting the ^{1}H-NMR assignments.

1. Iqbal, M.A.; Haque, R.A.; Ahamed, M.B.K.; Abdul, M.A.M.S.; Al-Rawi, S. Synthesis and anticancer activity of para-xylyl linked bis-benzimidazolium salts and respective Ag(I) *N*-heterocyclic carbene complexes. *Med. Chem. Res.* **2013**, *22*, 2455–2466.

2. Chauhan, H.P.S.; Carpenter, J.; Bhatiya, S.; Bakshi, A. Bis(Diethyldithiocarbamato)Antimony(III) Derivatives with Oxygen- and Sulfur-Donor Ligands: Synthesis, Esi-Mass, and Spectral Characterization. *Phosphorus Sulfur Silicon Relat. Elem.* **2013**, *188*, 1713–1722.
3. Goksel, F.S.; Bayrak, N.; Ibis, C. Synthesis of Novel *S*,*O*-Substituted 1,4-Benzoquinones. *Phosphorus Sulfur Silicon Relat. Elem*. **2013**, *189*, 113–123.
4. İbis, C.; Ayla, S.S.; Beyazit, N.; Bahar, H. Synthesis of Novel Thioethers and Sulfoxide Compounds. *Phosphorus Sulfur Silicon Relat. Elem*. **2013**, *188*, 1643–1651.
5. Gohari, A.R.; Hadjiakhoondi, A.; Sadat-Ebrahimi, E.; Saeidnia, S.; Shafiee, A. Cytotoxic terpenoids from Satureja macrantha CA Mey. *DARU J. Pharm. Sci*. **2005**, *13*, 177–181.
6. Sköld, M.; Karlberg, A.-T.; Matura, M.; Börje, A. The fragrance chemical β-caryophyllene—Air oxidation and skin sensitization. *Food Chem. Toxicol*. **2006**, *44*, 538–545.
